# Genes *mcr* improve the intestinal fitness of pathogenic *E. coli* and balance their lifestyle to commensalism

**DOI:** 10.1186/s40168-022-01457-y

**Published:** 2023-01-20

**Authors:** Dalmasso Guillaume, Beyrouthy Racha, Brugiroux Sandrine, Ruppé Etienne, Guillouard Laurent, Bonnin Virginie, Saint-Sardos Pierre, Ghozlane Amine, Gaumet Vincent, Barnich Nicolas, Delmas Julien, Bonnet Richard

**Affiliations:** 1 Université Clermont Auvergne, Inserm U1071, USC-INRAe 2018, Microbes, Intestin, Inflammation et Susceptibilité de l’Hôte (M2iSH), Centre de Recherche en Nutrition Humaine Auvergne, 28 place Henri Dunant, 63001 Clermont-Ferrand, France; 2 Centre de référence de la résistance aux antibiotiques, Centre Hospitalier Universitaire, 58 place Montalembert, 63000 Clermont-Ferrand, France; 3 Université de Paris, IAME, INSERM, F-75018 Paris, France; 4 AP-HP, Hôpital Bichat, DEBRC, F-75018 Paris, France; 5 Hub de Bioinformatique et Biostatistique—Département Biologie Computationnelle, Institut Pasteur, USR 3756 CNRS, Paris, France; 6 IMOST, UMR 1240 Inserm, Université Clermont Auvergne, 58 Rue Montalembert, 63005 Clermont-Ferrand, France

**Keywords:** *mcr-1*, Colistin, Antibiotic resistance, Antimicrobial peptides, Virulence, Inflammation, Microbiota, *Escherichia coli*, Commensalism

## Abstract

**Background:**

The plasmid-mediated resistance gene *mcr-1* confers colistin resistance in *Escherichia coli* and paves the way for the evolution to pan-drug resistance. We investigated the impact of *mcr-*1 in gut colonization in the absence of antibiotics using isogenic *E. coli* strains transformed with a plasmid encoding *or* devoid of *mcr-1*.

**Results:**

In gnotobiotic and conventional mice, *mcr-1* significantly enhanced intestinal anchoring of *E. coli* but impaired their lethal effect. This improvement of intestinal fitness was associated with a downregulation of intestinal inflammatory markers and the preservation of intestinal microbiota composition. The *mcr-1* gene mediated a cross-resistance to antimicrobial peptides secreted by the microbiota and intestinal epithelial cells (IECs), enhanced *E. coli* adhesion to IECs, and decreased the proinflammatory activity of both *E. coli* and its lipopolysaccharides.

**Conclusion:**

Overall, *mcr-1* changed multiple facets of bacterial behaviour and appeared as a factor enhancing commensal lifestyle and persistence in the gut even in the absence of antibiotics.

Video Abstract

**Supplementary Information:**

The online version contains supplementary material available at 10.1186/s40168-022-01457-y.

## Background

Antibiotic colistin has been reintroduced in recent years as an antibiotic of ‘last resort’ for treating infections caused by multidrug-resistant Gram-negative pathogens [1]. Its broad use has increased the prevalence of colistin resistance [2] and resulted in the identification of the first plasmid-mediated resistance gene *mcr-1* in extensively-drug resistant *Escherichia coli* in 2015 [3–5]. This acquired gene encodes a phosphoethanolamine transferase that modifies the charge of lipopolysaccharides (LPS) [6, 7] and, consequently, induces resistance to colistin-mediated bacterial lysis [8]. Thus far, 10 *mcr* genes and point variants have been reported [9]. Consequently, these *mcr* genes have been reported as an acquired resistance mechanism, paving the way for the evolution to toto-drug resistance. Their association with *E. coli* is a major source of concern since *E. coli* is a pathogenic symbiont of the intestinal microbiota, which can serve as a large reservoir of *mcr* genes. Accordingly, *E. coli* harbouring a plasmid-encoding *mcr*-1 have been observed in the digestive tract of humans, migratory birds, pets, and food animals [6, 10–13].

However, several elements suggest that *mcr* genes may be involved in functions that are not related to antibiotic resistance. They lead to a moderate increase in the colistin minimal inhibitory concentration (MIC) (≤8 μg/mL) compared to other resistance mechanisms conferring ≥64 μg/mL MIC values, like the *mgrB* inactivation in *Klebsiella pneumoniae*, thereby suggesting a low specificity for colistin. Found on a broad range of plasmids, *mcr-1* has been observed in bacteria susceptible to antibiotics and plasmids harbouring no other known resistance genes [6, 14], while ‘last-resort’ antibiotic resistance mechanisms are usually clustered in multiple-drug resistance plasmids [15, 16]. In addition, in an insect model of infection, *mcr-1* overexpression in a laboratory *E. coli* strain was found to decrease bacterial growth, the strain’s ability to activate macrophages, and its lethal activity [17], thereby suggesting that *mcr-1* has a broad impact on the physiology of *E. coli* and could play additional roles apart from colistin resistance*.*

In this study, we investigated the ability of *mcr-1*-harboring *E. coli* to colonize the gut in a gnotobiotic and conventional mouse model. For this purpose, we used the extraintestinal pathogenic *E. coli* reference strain CFT073 transformed by an empty plasmid (designated as *E. coli mcr-1*^*-*^) or its isogenic variant encoding *mcr-1* (designated as *E. coli mcr-1*^*+*^). We found that *mcr-1* affects multiple facets of host-bacteria interactions and promotes discreet encroaching of *E. coli* in the intestinal microbiota.

## Methods

### Bacterial strains and plasmids

To perform isogenic comparisons, the reference *E. coli* strain CFT073 [18] and the commensal *E. coli* strain HS were transformed with the kanamycin-resistant pBK-CMV empty vector (resulting in the strain referred to as *E. coli mcr*^*-*^) or a variant of pBK-CMV in which the *mcr-*1, *mcr*-3, -4, *mcr*-5, or *mcr*-10-encoding sequences were cloned downstream of the *lac* promoter (resulting in *E. coli mcr*^*+*^). To create rifampicin- or nalidixic acid-resistant bacteria, *E. coli* CFT073 was cultured overnight in Luria-Bertani (LB) medium and spread on LB agar plates containing rifampicin or nalidixic acid (50 μg/ml). One resistant clone was transformed using the pBK-CMV empty vector or pBK-CMV-*mcr-1* vector. The presence of *mcr-1* and its expression in the absence of a *lac* inducer resulted in colistin MIC values of 4 μg/mL, as usually observed in clinical isolates [6] and did not affect the growth of bacteria at 12 h (as well as up to at least 24 h) post-incubation in LB, Dulbecco’s modified Eagle medium (DMEM; Gibco, Waltham, MA, USA), or Roswell Park Memorial Institute 1640 (RPMI; Gibco) medium at 37°C (Supplementary Fig. 1). No inducer of the *lac* promoter was used in the experiments, and all experiments were performed with bacteria collected after 16 h of incubation at 37°C in LB medium.

The stability of pBK-CMV plasmid was assessed in *E. coli mcr*^*-*^ and *E. coli mcr*^*+*^ by serial propagation for 10 days in LB broth medium at 37°C without kanamycin. The passages were performed every day by diluting the culture 1:1000 in 10 ml sterile LB medium. Serial dilutions were spread daily on LB agar plates with or without kanamycin and the number of CFUs was counted to assess the loss of plasmids. The data show that the loss of empty or *mcr-1*-encoding pBK-CMV is similar. Even after 10 days of intensive growth without antibiotic, ~25% of *E. coli* population conserved the plasmid (Supplementary Fig. 2). Throughout this work, CFUs were counted on plates supplemented with kanamycin (pBK-CMV antibiotic resistance) to target *E. coli*-containing pBK-CMV plasmids.

For experiments using bacterial supernatants, bacteria were centrifuged for 10 min at 5500 rpm, and the supernatant was collected and then sterilized using a 0.22-μm filter. In addition, pBR plasmid-encoding colicin E1, E2, and D as well as pLR1 plasmid-encoding colicin A were hosted in the laboratory *E. coli* strain C600 (kindly provided by Dr. Denis Duché, UMR7255 CNRS-Aix-Marseille Université).

### Cloning of mcr genes

Genes *mcr-1*, *mcr-3*, *mcr-4*, *mcr-5,* and *mcr-10* were amplified using the GoTaq DNA polymerase (Promega) and cloned in the pBK-CMV plasmid (*mcr-3*, *mcr-4*, *mcr-5*, and *mcr-10*) with the following primers: *mcr1*EcoRI_For_ 5’-GCGAATTCATGATGCAGCATACTTCTGTG-3’, *mcr1*XhoI_Rev_ 5’-GTTCTCGAGTCAGCGGATGAATGCGGTG-3’; *mcr3*BamHI_For_ 5’-CGGGATCCATGCCTTCCCTTATAAAAAT-3’, *mcr3*EcoRI_Rev_ 5’-CGGGGAATTCTTATTGAACATTACGACATTG-3’; *mcr4*BamHI_For_ 5’-GCGGATCCGTGATTTCTAGATTTAAGACG-3’, *mcr4*EcoRI_Rev_ 5’-GCAGAATTCCTAATACCTGCAAGGTGC-3’; *mcr5*BamHI_For_ 5’-GCGGATCCATGCGGTTGTCTGCATTTATCAC-3’, *mcr5*EcoRI_Rev_ 5’-GCGAATTCTCATTGTGGTTGTCCTTTTCTGC-3’; *mcr10*BamHI_For_ 5’- GCGGATCCATGCCCGTACTTTTCAGGATG-3’, *mcr10*EcoRI_Rev_ 5’-GCGAATTCCTATCCACGACATTCGCGGAAC-3’. Empty or *mcr*-containing pBK-CMV plasmids were electroporated into *E. coli* strains and selected using kanamycin (50 μg/ml). The *mcr* gene sequences were double-checked by sequencing (GATC, Konstanz, Germany).

### Inactivation of Mcr-1 catalytic site

To inactivate the Mcr-1 enzyme produced from pBK-CMV, the threonine at position 285 that is involved in the binding of the active Zn atoms in the Mcr-1 catalytic site [19] was replaced by an alanine by site-directed mutagenesis using the InFusion HD Cloning Kit (Takara, Kusatsu, Japan), the Platinum SuperFi II DNA Polymerase (Invitrogen, Waltham, MA, USA), and T285A_For_ 5’-ATACGCCGCCGATGTGCCGCACGATGTG-3’, and T285A_Rev_ 5’-ACATCGGCGGCGTATTCTGTGCCGTG-3’ primers. The *mcr* gene sequence was double-checked by double-strand sequencing (GATC, Konstanz, Germany).

### Antimicrobial assays

Bacteria were grown overnight at 37°C on LB containing kanamycin (50 μg/ml). They were washed with 10-mM phosphate buffer (pH 7.2), suspended in 100 μl to obtain an optical density of 0.5 MacFarland, and then incubated for 2 h with antimicrobial peptides (1 μg for hBD1 and hBD2, 0.2 μg for hBD3 and LL37, 2 μg for HD-5, and 4 μg for HD-6). The bacteria were then spread on LB agar plates and incubated overnight at 37°C, and the CFUs were counted. All peptides used were obtained from the Peptide Institute, Osaka, Japan.

### Bacteriocin resistance assays

The antibacterial activity of bacteriocins against *E. coli mcr-1*^*+*^ and *E. coli mcr-1*^*-*^ was investigated in agar medium by an antibiogram-like approach as previously described [20]. Briefly, a bacterial suspension of 0.5 MacFarland was prepared from an overnight agar plate culture and diluted in sterile broth to obtain a final inoculum of approximately 10^5^ CFU/ml. After inoculation of Mueller-Hinton (MH) agar culture medium with this suspension, 10 μl of colicin-producing *E. coli* was spotted on the MH agar plate. After overnight incubation at 37°C, the plates were examined for colicin sensitivity, which was observed as clear zones of lysis in the overlaid strains. The inhibitory activity of colicins was then quantified in LB broth, as previously reported [20]. Briefly, colicin-producing *E. coli* and *E. coli mcr-1*^*+*^ or *E. coli mcr-1*^*-*^ at a 100:1 ratio were co-cultivated in 10 ml of LB broth at 37°C in a shaking incubator. After 24 h of incubation, serial dilutions of cocultures were spread on blood sheep agar plates (bioMérieux, France), and the CFUs of *E. coli* CFT073 *mcr-1*^*+*^ and *E. coli* CFT073 *mcr*^*-*^—which have a hemolysin phenotype (unlike *E. coli* C600)—were counted.

### LPS purification

LPS was purified as previously described [21]. Briefly, bacteria were grown overnight at 37°C in 50 ml of LB containing kanamycin (50 μg/ml). They were collected by centrifugation, washed twice with phosphate-buffered saline (PBS, Gibco) containing 0.15 mM CaCl_2_ and 0.5 mM MgCl_2_, and then disrupted by sonication. To eliminate the remaining nucleic acids and proteins, lysates were treated with 200 μg/ml proteinase K (1 h at 65°C with gentle mixing) and then with 40 μg/ml DNase and 80 μg/ml RNase (37°C, in the presence of 1 μl/ml 20% MgSO_4_ and 4 μl/ml chloroform overnight with gentle mixing). Finally, an equal volume of hot (68°C) 90% phenol was added to the mixtures, followed by vigorous shaking at 68°C for 15 min. Suspensions were then cooled on ice and centrifuged at 8500 rpm for 15 min. Aqueous phases were pooled, and phenol phases were re-extracted with 10 ml of distilled water at 68°C. Pooled aqueous phases were extensively dialyzed against distilled water at 4°C, and the purified LPS product was finally lyophilized. For experiments, LPS was dissolved in PBS.

### Cell culture

The human intestinal epithelial HT-29 (ATCC® HTB-38™) and the human monocyte THP-1 (ATCC® TIB-202™) cell lines were maintained in an atmosphere containing 5% CO_2_ at 37°C in the culture media recommended by ATCC. The human mucus-producing intestinal epithelial HT29-16E cell line [22] were grown in Dulbecco’s modified Eagle’s medium supplemented with 10% (v/v) fetal calf serum (Lonza), 1% l-glutamine (Life Technologies), 200 U penicillin, 50 mg of streptomycin, and 0.25 mg of amphotericin B per litre. In addition, THP-1 monocytes were differentiated into macrophages by treatment with 20 ng/ml phorbol myristate acetate (PMA) for 18 h.

### Cell infection

Cells were infected with a multiplicity of infection (MOI) of 100 (HT-29) or 10 (THP-1) for the indicated time. To determine bacterial adhesion, cells were extensively washed with sterile PBS, and serial dilutions were spread on LB agar plates. After one night at 37°C, the number of CFUs was counted.

### Immunofluorescence staining

HT29-16E cells were seeded on sterile glass cover slips. After 21 days of culture, the cells were infected at MOI of 100 for 30 min. After several washes, cells were fixed, incubated at room temperature in the hybridization buffer (20 mM Tris-HCl (pH 7.8), 1.25 M NaCl, 0.01% SDS, Denhardt’s solution 1X), and then incubated for 3 h with the DNA probe (5 ng/μL; 5’-GCA AAG GTA TTA ACT TTA CTC TTC TCC-Cy2-3’). Cells were washed with hot hybridization buffer (48°C) and stained for 10 min using DAPI (1 μg/mL) and WGA (1 μg/mL). Cells were washed 3 times and observed using a Zeiss LSM 800 confocal microscope. Bacteria were counted using image analysis software Imaris.

### Enzyme-linked immunosorbent assays (ELISAs)

The amounts of KC, IL-1β, IL-6, IL-8, and TNF-α secreted in the cell culture supernatants or present in mouse tissues and lipocalin-2 present in mouse faeces were determined by ELISA (R&D Systems) in accordance with the manufacturer’s instructions.

### Mouse sensitivity to *E. coli* mcr-1^+^ or *E. coli* mcr-1^-^ and their derivate LPS

Six- to 8-week-old C57/BL6 male mice (Charles River, Ecully, France) received an intraperitoneal administration of 5 mg/ml LPS or 10^9^ CFUs of bacteria in 0.2 ml of PBS.

### *E. coli* mcr-1^+^ or *E. coli* mcr-1^-^ infection using the oligo-mouse-microbiota 12 (OMM^12^) model

OMM^12^ (C57Bl/6J) mice are gnotobiotic mice that harbor a defined consortium of 12 bacterial strains isolated from the murine gut and are devoid of *E. coli* strains (see Table S1) [23]. These mice are also designated stable defined moderately diverse microbiota mice (sDMDMm2) [24]. OMM^12^ mouse breeding pairs were kindly provided by Dr. Basic and Prof. Bleich (Hannover Medical School, Institute for Laboratory Animal Science, Hannover, Germany) and bred under germ-free conditions in a flexible isolator at the INRAe mouse facility (UMR454 MEDIS, Theix, France). Mouse colonies were regularly checked for potential contaminants using qPCR and culture-based approaches.

OMM^12^ mouse experiments were performed using an individually ventilated and positively pressurized cage system (IsoCage P—bioexclusion system, Tecniplast, France). Each experiment was performed using either 8- or 18-week-old littermates subjected to 12:12 light/dark cycles with access to food and water *ad libitum*. Briefly, OMM^12^ mice were transferred into the IsoCage P system 2 days prior to the beginning of the experiment to allow for the gut microbiota to stabilize. Further, the mice were orally infected with 10^3^ bacteria/mouse resuspended in 50 μl of PBS and then sacrificed 10 days post-infection by cervical dislocation.

### *E. coli* mcr-1^+^ and *E. coli* mcr-1^-^ infection using a conventional mouse model

Experiments were performed using wild-type or *Camp* knock-out six- to 8-week-old C57/BL6 male mice (Charles River and The Jackson Laboratory, respectively) housed in a specific pathogen-free animal facility at the University of Clermont Auvergne, France. Mice were fed standard chow ad libitum throughout the experiments, had free access to sterile water, and were subjected to 12:12 light/dark cycles. In addition, they received oral gavage with a 200-μl suspension containing 10^8^ CFUs of *E. coli mcr-1*^*+*^ or *E. coli mcr-1*^*-*^ and then sacrificed 3 days post-infection by cervical dislocation.

For the competition experiments, mice received streptomycin in drinking water (2.5 g/L) for 48 h. The antibiotic was then discontinued, and 24 h later, the mice received 10^8^ CFUs of *E. coli mcr-1*^*+*^ (rifampicin resistant strain) and 10^8^ CFUs of *E. coli mcr-1*^*-*^ (nalidixic acid-resistant strain) in 200 μl of PBS. At the indicated time, serial dilutions of feces were spread on LB agar plates containing kanamycin (50 μg/ml) and either rifampicin (50 μg/ml) or nalidixic acid (50 μg/ml) in order to count *E. coli mcr-1*^*+*^ and *E. coli mcr-1*^*-*^ CFUs, respectively.

### Quantification of *E. coli* mcr-1^+^ and *E. coli* mcr-1^-^ in mice feces

Fecal samples were collected at the indicated times, diluted in sterile PBS, and then plated on LB agar containing kanamycin (50 μg/ml) and either rifampicin (50 μg/ml) or nalidixic acid (50 μg/ml) to quantify the number of *E. coli mcr-1*^*+*^ and *E. coli mcr-1*^*-*^. Bacterial identification was performed using chromogenic medium or mass spectrometry, and the presence of *mcr-1* in isolates was confirmed by PCR. No such resistant or *mcr-1*-positive bacteria were found in uninfected mice. Intestinal tissues were longitudinally opened, washed in 3 ml of sterile PBS, and then either homogenized using a Tissue Master 125 Homogenizer (Omni International) to quantify *E. coli* tissue-associated loads or directly incubated in sterile DMEM containing antibiotics to quantify the secreted cytokines.

### Quantitative (q)PCR of bacterial 16S rRNA genes

Fecal DNA extraction was performed as described in Herp et al. (2019), with the only difference being that DNA was diluted to a final concentration of 10 ng/μl in dH_2_O [25]. The DNA concentration was determined using a Qubit 3 fluorometer and its corresponding kit (Qubit® dsDNA HS Assay kit (0.2–100 ng), Thermo Fisher Scientific). Absolute 16S rRNA gene quantification was performed as described in Brugiroux et al. (2016), with modifications [23]. In addition, QPCR assays were run and analysed on a CFX96 Touch Real-Time PCR Detection System (Bio-Rad). All qPCRs were performed using 60°C as the optimal annealing temperature, except for the qPCR detection of the I48 strain, which was performed using 54°C to optimize amplification efficiency. Detection of *E. coli* was assessed using the optimal OMM^12^ qPCR program (95°C for 10 min, followed by 45 cycles of 95°C for 15 s and 60°C for 1 min), with *E. coli* forward (5’-CATGCCGCGTGTATGAAGAA-3’) and reverse (5’-CGGGTAACGTCAATGAGCAAA-3’) primers and the corresponding probe (Taqman FAM-labeled probe 5’-TATTAACTTTACTCCCTTCCTCCCCGCTGAA-3’) published by Huijsdens et al. [26].

### Sequencing of the bacterial 16S rRNA genes

Fecal samples were immediately frozen in liquid nitrogen after defecation and stored at −80°C. Within 1 month, DNA was extracted from the fecal samples according to the International Human Microbiome Standards protocol, as previously described [27]. A negative control devoid of DNA as well as bacterial and DNA-positive controls were also processed throughout the entire experiment (ZymoBIOMICS®, Zymo Research). The genomic DNA was amplified with fusion primers targeting the variable V3 and V4 regions of the 16S rRNA gene with indexing barcodes, as previously described [28]. All samples were pooled for 2 x 300 bp paired-end sequencing on the Illumina MiSeq platform (Illumina), in accordance with the manufacturer’s specifications, at the Clermont-Ferrand University Hospital, France. The reads were deposited in the European Nucleotide Archive (project ID: PRJEB33293).

### Microbiota composition analysis

Paired-end read assembly, quality and length filtering, OTU picking (100% sequence identity threshold), and chimera removal were performed with UPARSE [29]. After quality-filtering and trimming, an average of 26,175 sequences were acquired for each sample. Allele-specific variants were assigned to taxonomy by QIIME 2 (https://qiime2.org/) with the SILVA database (version 132, https://www.arb-silva.de/). Sequence counts were normalized to their sample size and then multiplied by the size of the smaller sample (*n* = 5,500). Sequences that were not observed more than three times in at least 15% of the samples (*n* = 4) were discarded.

The statistical analyses were performed in R (https://www.r-project.org/), with vegan (https://CRAN.R-project.org/package=vegan) and phyloseq packages [30]. The Kruskal-Wallis test was used to estimate alpha diversity differences among groups, and pairwise comparison was performed with the Wilcoxon test with a correction of *p* values in accordance with the FDR procedure. Beta diversity was assessed from Jensen-Shannon and generalized UniFrac indices, which were reported after non-metric multidimensional scaling (NMDS). Adonis (PERMANOVA) and PermDISP2 tests were used to assess significant differences among groups. Significant differences in taxon abundances between groups were detected with the DEseq2 approach and correction of *p* values according to the FDR procedure.

### Pairwise comparative modelling (PCM)

PCM was used to predict *mcr*-*1* analogues in the intestinal microbiota, as previously described [31]. PCM is based on using homology modeling to increase the specificity of the functional prediction of proteins, particularly when they are distantly related to potential homologs. Taxonomy was assigned, as previously described [31], by combining results obtained from a BLASTN against the National Centre for Biotechnology Information (NCBI) genome database (minimal 70% identity and 80% coverage), a BLASTN against the IMOMI in-house database (minimal 85% identity and 90% coverage), and the taxonomy of the metagenomic unit whenever applicable.

### Ethical statement

Animal protocols were in accordance with the recommendations of the Guide for the Care and Use of Laboratory Animals of the University of Clermont Auvergne and were approved by the French Ministry of National Education, Higher Education, and Research (APAFIS#16354, #22507, #22770).

### Statistical analysis

Values are expressed as means ± SEMs. Statistical tests were performed with GraphPad Prism version 6.07 software, using a two-tailed Student’s *t* test or a Mann-Whitney *U* test depending on normality determined using the D’Agostino-Pearson omnibus normality test. *P* values less than 0.05 were considered statistically significant.

## Results

### Mcr-1 enhances gut bacterial encroachment in the oligo-mouse-microbiota 12 model

**Fig. 1:**
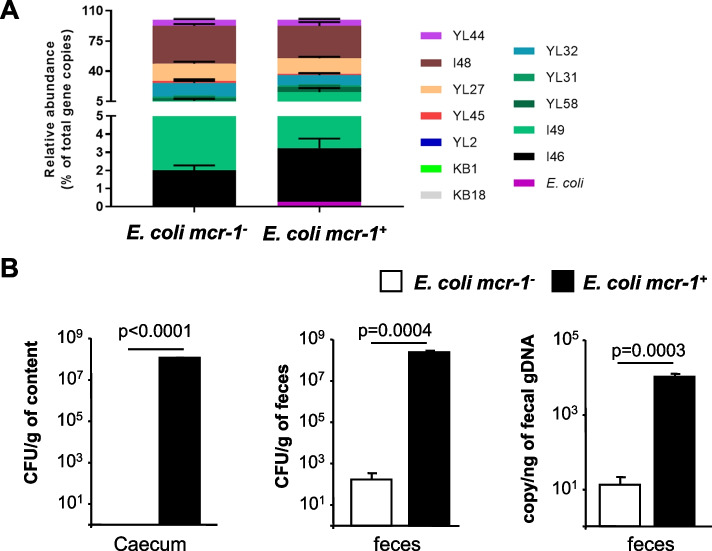
*mcr-1* enhances gut colonization in the oligo-mouse-microbiota 12 (OMM^12^) model without drastically affecting gut microbiota composition. Gnotobiotic OMM^12^ mice were orally infected with *E. coli mcr-1*^*+*^ or with *E. coli mcr-1*^*-*^*,* and intestinal colonization was assessed at day 10 post-infection. **A** The relative abundances of OMM^12^ strains were determined by a strain-specific qPCR assay and are plotted as the relative abundances (expressed as the fraction of total 16S rRNA gene copy numbers). YL44, *Akkermansia muciniphila*; I48, *Bacteroides caecimuris*; YL27, *Muribaculum intestinale*; YL45, *Turicimonas muris*; YL2, *Bifidobacterium longum*; KB1, *Enterococcus faecalis*; KB18, *Acutalibacter muris*; YL32*, Clostridium clostridioforme*; YL31, *Flavonifractor plautii*; YL58, *Blautia coccoides*; I49, *Lactobacillus reuteri*; I46, *Clostridium innocuum*; and *E. coli* CFT073. Data are means ± SEMs. **B ***E. coli* abundance was assessed in caecum content and faeces by the serial dilution culture method (expressed as CFUs per g of caecum content or faeces) and by *E. coli*-specific 16S rRNA gene qPCR (expressed as copy numbers per ng of fecal genomic DNA). Data are means ± SEMs

To investigate the role of *mcr-1* in gut colonization, we used the OMM^12^ model, in which gnotobiotic mice are colonized by a stable microbiota of 12 sequenced strains representing the major bacterial phyla in the murine gut without the presence of Proteobacteria like *E. coli* [23]. The mice were orally infected with a low bacterial inoculum (10^3^ colony-forming units [CFU]) of *E. coli* CFT073 *mcr-1*^*+*^ or E*. coli mcr-1*^*-*^*.* Mice were killed at day 10 post-infection. Inoculated bacteria were quantitated in faeces—which reflects the levels of colonization of the intestine—and inflammation status was assessed in faeces and intestinal tissues. Successful colonization of the mouse gut by the oligo mouse microbiota was confirmed by 16S rRNA gene qPCR assay (Fig. 1A). Compositional analysis indicated successful encroachment of OMM^12^ taxa and only subtle differences in the abundance of bacteria between mice infected by *E. coli mcr-1*^*+*^ and E*. coli mcr-1*^*-*^ (Fig. 1A and Supplementary Fig. 3). Unlike *E. coli mcr-1*^*-*^ cells, *E. coli mcr-1*^*+*^ were detected in the cecal content (Fig. 1B). From feces, 16S rRNA gene qPCR assay and serial dilution cultures revealed a significant >1000-fold increase in *E. coli mcr-1*^*+*^ loads compared to *E. coli mcr-1*^*-*^ loads (Fig. 1B). The secretion of CXCL1/KC (the murine analogue of human IL-8), IL-1β, and IL-6 cytokines was measured in colonic tissues to assess gut inflammatory status in response to colonization by *E. coli mcr-1*^*+*^ or by *E. coli mcr-1*^*-*^ (Supplementary Fig. 4A–C). Despite a major difference in terms of gut colonization between *E. coli mcr-1*^*+*^ and *E. coli mcr-1*^*-*^, cytokine expression was not significantly different in mice colonized by *E. coli mcr-1*^*+*^ compared to mice colonized by *E. coli mcr-1*^*-*^. Furthermore, the concentration of lipocalin-2 in feces, which is a sensitive marker of intestinal inflammation [32], was lower in mice infected with *E. coli mcr-1*^*+*^ than in mice infected with *E. coli mcr-1*^*-*^, but it was not significantly different (Supplementary Fig. 4D). Overall, the results suggest that *mcr*-1 enhances gut colonization in the OMM^12^ model without affecting gut inflammatory status.

### mcr-1 enhances gut colonization and preserves microbiota composition in conventional mice

**Fig. 2:**
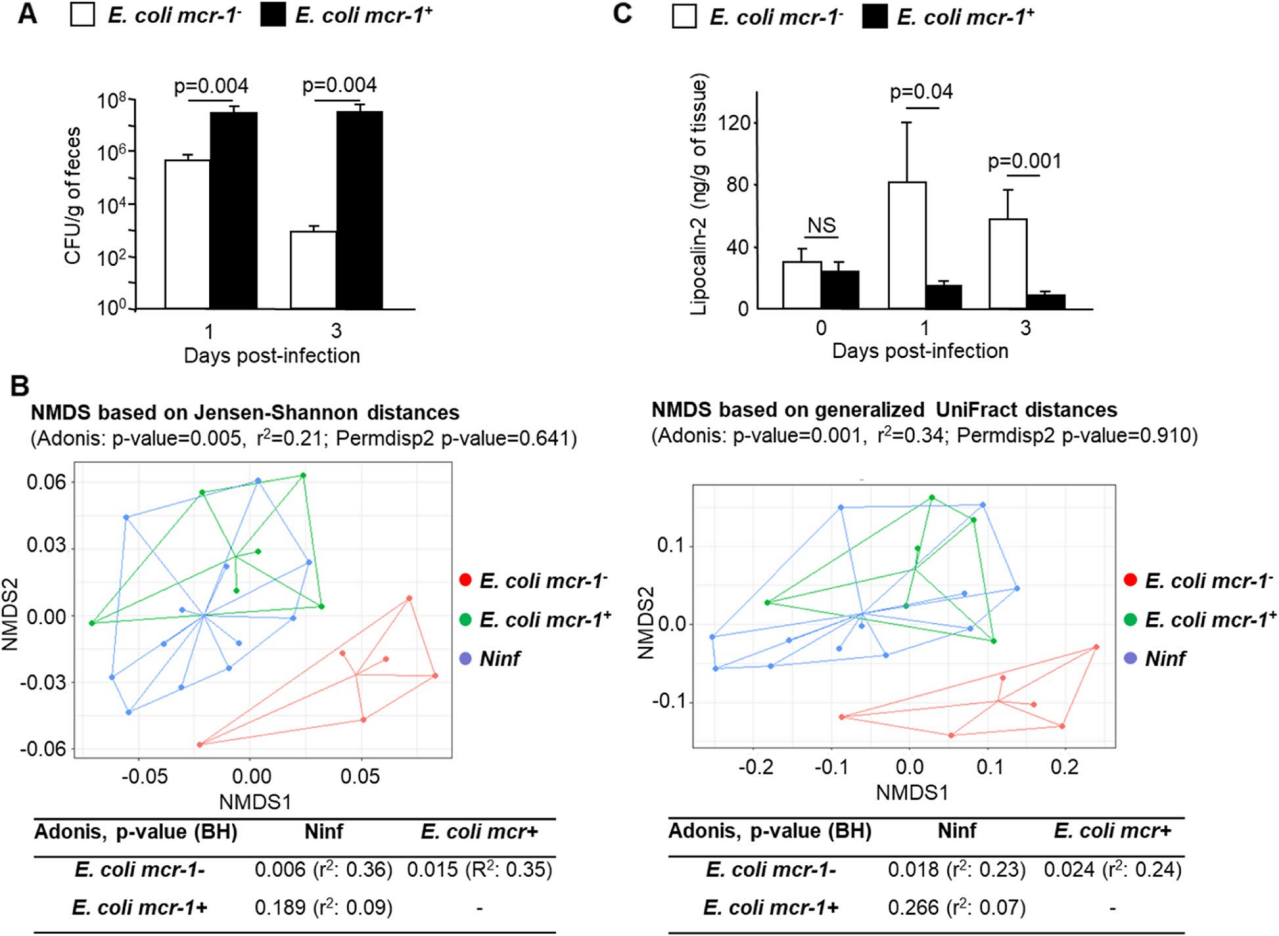
*mcr-1* promotes colonization of the mouse intestinal tract, preserves microbiota composition, and reduces inflammation. Mice were challenged by oral gavage with *E. coli mcr-1*^*+*^ or with *E. coli mcr-1*^*-*^. **A** The numbers of *E. coli mcr-1*^*+*^ or *E. coli mcr-1*^*-*^ were determined from the feces. Data are represented as means ± SEMs. **B** Intestinal microbiota composition was assessed from faecal 16S rRNA gene sequencing before *E. coli mcr-1*^*+*^ or *E. coli mcr-1*^*-*^ inoculation (Ninf) and at 3 days post-infection. The difference in composition measured by Jensen-Shannon and generalized UniFrac metrics are presented as nonmetric multidimensional scaling (NMDS) ordinations. In addition, Adonis tests were performed to assess significant differences in intestinal microbiota structure, and PermDisp2 was used to assess significant differences in dispersion within sample groups. The *p* values were adjusted for multiple group comparisons by the Benjamini-Hochberg (BH) procedure. **C** Fecal lipocalin-2 levels were measured by ELISA. Data are represented as means ± SEMs. NS indicates ‘not significant’

To confirm the results obtained using the OMM^12^ model in a more physiological context, conventional mice were orally infected with 10^8^ CFUs of *E. coli mcr-1*^*+*^ or *E. coli mcr-1*^*-*^. Feces were collected before inoculation as well as at day 1 and day 3 post-inoculation. Furthermore, colonic and ileal tissues were collected after animal sacrifice. As expected, *E. coli* CFT073 was not detected in mouse feces before inoculation (data not shown). After *E. coli* CFT073 inoculation, *E. coli mcr-1*^*+*^ recovered at higher rates than *E. coli mcr-1*^*-*^*,* and the differences in the intestinal colonization levels increased at day 3 post-inoculation (Fig. 2A). Hence, *mcr*-1 promotes the encroachment of *E. coli* CFT073 in the gut and enhances its persistence, as confirmed in a competition experiment, since *E. coli mcr-1*^*+*^ were found in a higher number than *E. coli mcr-1*^*-*^ in mice feces after co-infection (Supplementary Fig. 5).

Further, the intestinal microbiota composition of conventional mice was analyzed before and after colonization by *E. coli* CFT073. The diversity of the intestinal microbiota was assessed by four metrics (supplementary Fig. 6). The Shannon index, InvSimpson index, and evenness index did not reveal any significant differences among the three mouse groups. However, diversity assessed from the richness index was significantly lower in mice inoculated with *E. coli mcr-1*^*-*^ than in non-infected mice and mice inoculated with *E. coli mcr-1*^*+*^ (Kruskal-Wallis test, *p* value = 0.01; pairwise Wilcoxon test, adjusted *p* values: 0.013 and 0.015, respectively). In contrast, no difference in richness was observed between non-infected mice and mice inoculated with *E. coli mcr-1*^*+*^ (pairwise Wilcoxon test, adjusted *p* value = 0.56). Thereafter, the differences in microbiota structure between the three mouse groups were assessed by the Jensen-Shannon distance (JSD) and the generalized UniFrac distance (Fig. 2B). For both metrics, PermDISP2 tests revealed similar dispersions of the microbiota compositions within the mouse groups (*p* values: 0.641–0.910). However, the microbiota structure of mice colonized by *E. coli mcr-1*^*-*^ differed significantly from those of non-infected mice and mice colonized by *E. coli mcr-1*^*+*^ (Adonis, adjusted *p* values: 0.006–0.024), which had indistinguishable intestinal microbial communities (Adonis, adjusted *p* values: 0.186–0.266). These results were consistent with non-metric multidimensional scaling (NMDS) ordinations in which samples showed similar dispersions and were consistently separated according to the *mcr*-1 status (Fig. 2B). Accordingly, 10 bacterial taxa were identified at the genus level as differentially abundant in mice inoculated with *E. coli mcr-1*^*-*^-compared with non-infected mice or mice inoculated with *E. coli mcr-1*^*+*^ (supplementary Fig. 7). Between these latter two groups, there was no significant difference in terms of bacterial abundance.

Overall, these results revealed that *mcr*-1 prevents alterations of microbiota composition caused by *E. coli* CFT073 intruding in the gut and, thereby, promotes its discreet encroachment and persistence.

### mcr-1 prevents intestinal inflammation

To assess the inflammatory status of the intestinal tract in response to colonization by *E. coli mcr-1*^*+*^, the secretion of KC, IL-1β, IL-6, and TNF-α cytokines was measured in ileal and colonic tissues of conventional mice (supplementary Fig. 8). The KC and IL-6 secretion rates in the ileum and the colon were significantly lower in mice colonized by *E. coli mcr-1*^*+*^ than in mice colonized by *E. coli mcr-1*^*-*^. Furthermore, the expression of TNF-α and IL-1β was not significantly modified in the colon (*p* values 0.65 and 0.48, respectively). In contrast, a significant decrease in IL-1β production was observed in the ileum (*p* value: 0.038). In addition, the concentration of lipocalin-2 in feces was also measured to strengthen the results (Fig. 2C). As expected, no significant difference in secretion was observed in the animals before bacterial inoculation. However, compared to inoculation with *E. coli mcr-1*^*-*^*,* inoculation of *E. coli mcr-1*^*+*^ induced a significant decrease in the production of lipocalin-2 at 1- and 3-days post-inoculation (*p* values, 0.04 and 0.001, respectively). The results were consistent with those obtained from gnotobiotic mice and confirmed those obtained for cytokine measurements from conventional mouse intestinal tissues.

Overall, these results reveal that *mcr-1* decreases *E. coli* pro-inflammatory activity during gut colonization.

### mcr-1 induces resistance to human and bacterial antimicrobial peptides

**Fig. 3:**
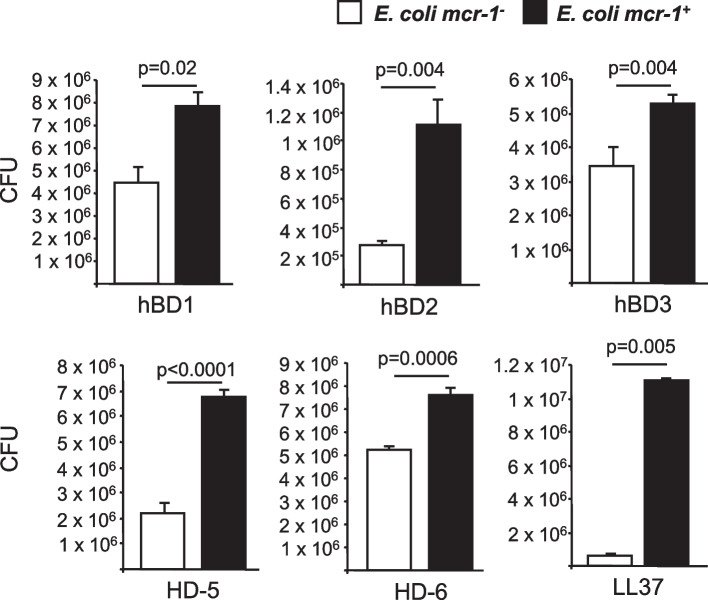
*mcr-1* increases bacterial resistance to human antimicrobial peptides. 2.10^7^*E. coli mcr-1*^*+*^ or 2.10^7^*E. coli mcr-1*^*-*^ were incubated for 2 h with α-defensins (hBD1 to 3), β-defensins (HD-5 and -6), and cathelicidin (LL-37). The numbers of surviving bacteria are reported as the number of colony-forming units (CFUs). Data are means ± SEMs of four replicates and are representative of three independent experiments

Since *mcr*-1 induces resistance to colistin, we investigated its role in resistance to structurally related antimicrobial peptides (AMPs) produced in the gut: β-defensins 1 to 3 (hBD) and cathelicidin LL37—which are produced by intestinal epithelial cells (IECs)—and Paneth cell-specific alpha-defensins 5 and 6 (HD) [33, 34]. AMPs hDB1–3, LL37, and HD-5 and HD-6 significantly decreased the growth of *E. coli mcr-1*^*-*^ compared to that of *E. coli mcr-1*^*+*^ (Fig. 3; *p* values, 0.02 to <0.0001), which indicates that *mcr*-1 increases *E. coli* resistance to AMPs. In order to investigate the impact of eukaryotic AMPs on *E. coli mcr-1*^*+*^ intestinal colonization, *Cramp* (the murine homolog of *LL-37*) knock-out mice were infected with *E. coli mcr-1*^*+*^ and *E. coli mcr-1*^*-*^. As depicted in supplementary Fig. 9, *E. coli-mcr-1*^*+*^ exhibited a better colonization of the gut compared to *E. coli-mcr-1*^*-*^*.* Overall, our results reveal that the *mcr*-1 gene induces a cross-resistance between colistin, and AMPs secreted by intestinal cells. However, AMP resistance is probably not the only feature involved in the increased intestinal fitness mediated by *mcr*-1.

**Fig. 4:**
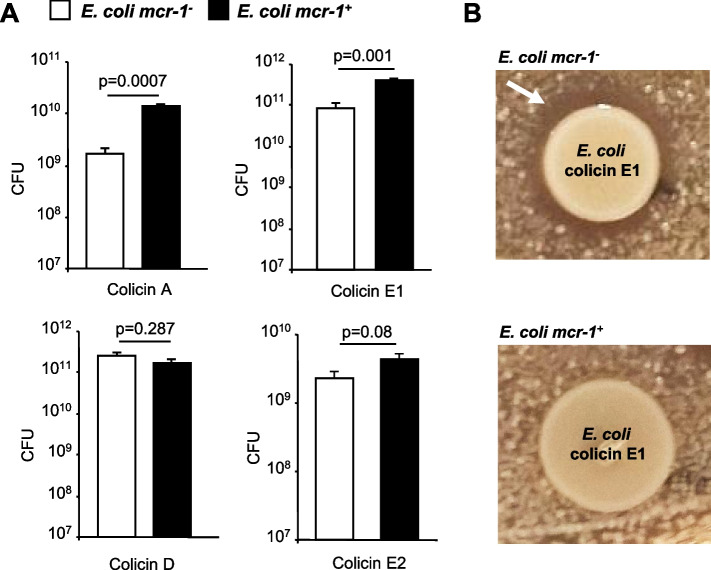
*mcr-1* increases bacterial resistance to colicin antimicrobial peptides. **A** Growth competition between *E. coli mcr-1*^*+*^ and *E. coli mcr-1*^*-*^ in the presence of *E. coli* C600 strains producing colicin A, D, E1, or E2. After 16 h of coculture in LB medium, serial dilutions were spread on blood sheep agar plates, and *E. coli mcr-1*^*+*^ and *E. coli mcr-1*^*-*^ colonies were counted. Data are expressed as the number of colony-forming units (CFUs). Data are means ± SEMs of four replicates and are representative of three independent experiments. **B ***E. coli mcr-1*^*+*^ or *E. coli mcr-1*^*-*^ were spread on MH agar, and 10 μl bacterial pellets of colicin-producing *E. coli* were spotted. Representative pictures are presented. The white arrow indicates the growth inhibition zone

Bacteria of the gut microbiota can also produce AMPs called bacteriocins, such as colicins E1 and A (which induce membrane permeabilization) and colicins E2 and D (which target nucleic acids) [35]. We investigated the ability of the *mcr-1* gene to affect the efficacy of such prevalent anti-competitor bacterial effectors (Fig. 4). Interestingly, it was found that laboratory *E. coli* C600 strains producing pore-forming colicins E1 and A inhibited the growth of *E. coli mcr-1*^*-*^, while *E. coli mcr-1*^*+*^ significantly resisted the inhibitory activity of those *E. coli* C600 strains producing membrane pore-forming colicins E1 and A (*p* values, ≤0.001). In contrast, the presence of *mcr*-1 did not significantly decrease the activity of E2 and D bacterial nucleases (*p* values 0.08–0.287).

Hence, *mcr*-1 decreases susceptibility to both human and bacterial AMPs produced in the gut by intestinal epithelial cells and microbiota.

### mcr-1 masks the pro-inflammatory activity of LPS and affect bacterial virulence

**Fig. 5:**
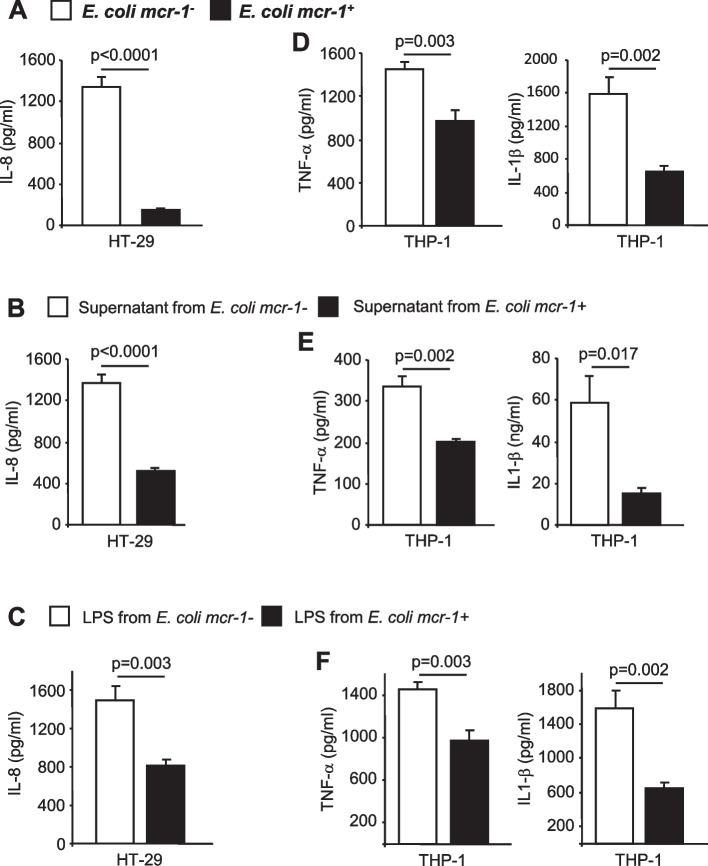
*mcr-1* decreases the pro-inflammatory response. **A** HT-29 cells were infected at an MOI of 100 for 3 h with *E. coli mcr-1*^*+*^ or with *E. coli mcr-1*^*-*^. HT-29 cells were stimulated with **B** bacterial culture supernatant (10% vol/vol) for 3 h or **C** stimulated for 3 h with LPS (10 μg/ml) purified from *E. coli mcr-1*^*+*^ or *E. coli mcr-1*^*-*^. **D–F** The same experiments as those in **A–C** were performed using THP-1 cells. **D** THP-1 cells were infected at a MOI of 10 for 1 h and **E** stimulated with 10% vol/vol bacterial supernatant for 1 h or **F** stimulated with 0.01 μg/ml LPS for 3 h. **A**–**F** The amounts of secreted IL-8, IL-1β, and TNF-α in the cell culture supernatant were quantified by ELISA. Data are the means ± SEMs of six replicates and are representative of three independent experiments

LPS, the target of *mcr*-1, is recognized by innate immune receptors and, consequently, promotes the expression of pro-inflammatory genes [36]. The resulting inflammation is responsible for septic shock but is also critical to host recovery. We investigated the pro-inflammatory responses of HT-29, and THP-1 cells infected with *E. coli mcr*-1^+^ or *E. coli mcr*-1^-^. Similar experiments were performed by incubating cells with bacterial culture supernatants and LPS purified from *E. coli mcr*-1^+^ or *E. coli mcr-*1^-^. We found that infection with *E. coli mcr*-1^+^ significantly decreased the secretion of pro-inflammatory cytokines IL-8, IL-1β, and TNF-α (Fig. 5A, D; *p* values, 0.002 to <0.0001). Similar results were observed with culture supernatants (Fig. 5B, E) and purified LPS (Fig. 5C, F) derived from *E. coli mcr-1*^*+*^. To strengthen these results, we next investigated whether *mcr*-1 modifies the virulence of *E. coli*. For this purpose, we used a sepsis model in which mice received an intraperitoneal administration of 10^9^*E. coli mcr-1*^+^ or *E. coli mcr-1*^-^. Administration of *E. coli mcr-1*^*-*^ resulted in 100% mortality at 48 h. All mice that were injected with *E. coli mcr-1*^+^ survived (*p* value: <0.0001, supplementary Fig. 10A). A similar trend was observed in intraperitoneal inoculation with purified LPS (*p* value: 0.1, supplementary Fig. 10B).

Overall, the results reveal that *mcr*-1 has a peacekeeper role in controlling the innate immune response that can impair gut colonization.

### mcr-1 enhances adhesion of *E. coli* to host cells

**Fig. 6:**
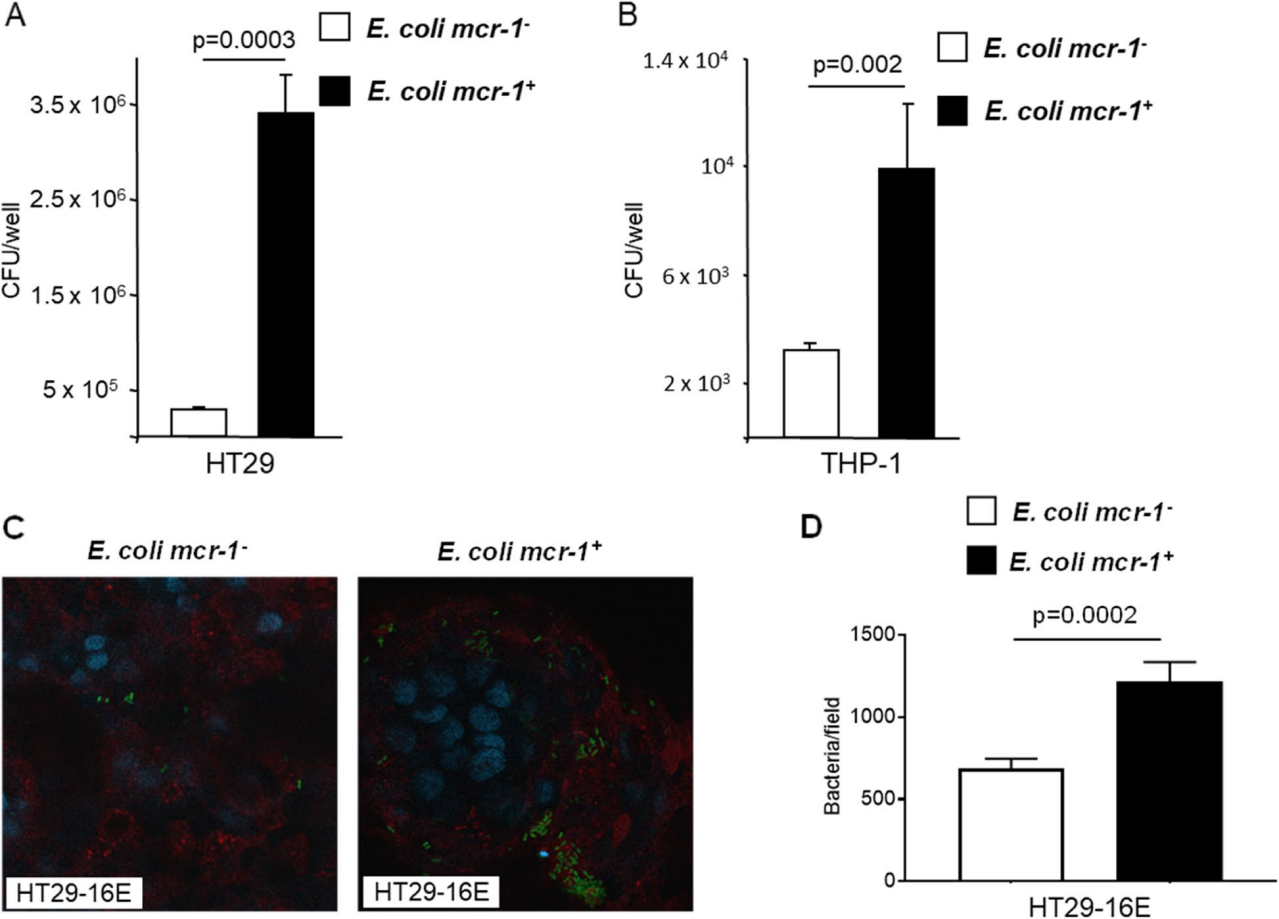
*mcr*-*1* increases bacterial adherence to eukaryotic cells. **A** HT-29 cells and **B** THP-1 cells were infected for 3 h and 1 h, respectively, with *E. coli mcr-1*^*+*^ or with *E. coli mcr-1*^*-*^. Adherent bacteria were counted, and the data are expressed as the number of colony-forming units (CFUs)/well. Data are the means ± SEMs of six replicates and are representative of three independent experiments. **C**, **D** HT29-16E cells were infected *E. coli mcr-1*^*+*^ or with *E. coli mcr-1*^*-*^ and stained for bacteria (red), mucus (green), and nuclei (blue). **C** Representative images are shown. **D** Quantification of bacteria per fields. Data are the means ± SEMs of 26 fields and are representative of two independent experiments

Modifications in LPS mediated by *mcr*-1 can affect the charge of bacterial outer membranes and membrane fluidity [37]. We therefore hypothesize that *mcr*-1 could affect bacterial adhesion to host cells. The adhesion of *E. coli mcr-1*^*+*^ and *E. coli mcr-1*^*-*^ to HT29 IECs and THP-1 macrophages was investigated (Fig. 6A and B, respectively). The adhesion of *E. coli mcr*-1^+^ to HT29 cells witnessed a ten-fold increase compared to the adhesion of *E. coli mcr*-1^-^ (*p* value, 0.0003); similar results were observed with THP-1 cells (*p* value: 0.002). In the gut, IECs harbor a mucus layer to limit bacteria-cell interactions. We infected the mucin-hyperproducing HT29-16E cells [22] and found that *E. coli mcr*-1^+^ were also able to significantly better adhere to mucus than *E. coli mcr*-1^-^ (Fig. 6C, D).

Therefore, the presence of *mcr*-1 enhances bacterial adhesion to mucus and host intestinal epithelial cells, a key function that is potentially involved in the promotion of bacterial gut colonization.

### Modifications induced by mcr-1 are observed in a commensal *E. coli* transformed by pBK-CMV-mcr-1

**Fig. 7:**
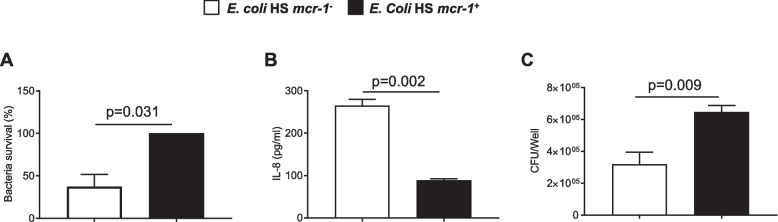
Expression of Mcr-1 by commensal *E. coli* HS strain, induces resistance to LL-37, decreases LPS pro-inflammatory response and increases bacterial adhesion to eukaryotic cells. *E. coli* HS were transformed with empty pBK-CMV (*E. coli mcr-1*^*-*^) or with pBK-CMV-*mcr-1* (*E. coli mcr-1*^*+*^). **A** 2.10^7^*E. coli mcr-1*^*+*^ or 2.10^7^*E. coli mcr-1*^*-*^ were incubated for 1.30 h with 0.1 μg of LL-37. The percentage of surviving bacteria was reported. Data are the means ± SEMs of four replicates and are representative of six independent experiments. **B** HT-29 cells were stimulated for 3 h with LPS (10 μg/ml) purified from *E. coli mcr-1*^*+*^ or *E. coli mcr-1*^*-*^. The amounts of secreted IL-8 in the cell culture supernatant were quantified by ELISA. Data are the means ± SEMs of six replicates and are representative of two independent experiments. **C** HT-29 cells were infected for 3 h with *E. coli mcr-1*^*+*^ or with *E. coli mcr-1*^*-*^. Adherent bacteria were counted, and the data are expressed as the number of colony-forming units (CFUs)/well. Data are the means ± SEMs of six replicates and are representative of two independent experiments

Since *E. coli* CFT073 is a pathogenic bacterium, we aimed at investigated the impact of *mcr-1* gene in the commensal *E. coli* HS strain. As expected, electroporation of pBK-CMV-*mcr-1* induced a MIC to colistin of 4 μg/mL versus 0.5 μg/mL for *E. coli* HS transformed with the empty plasmid (data not shown). *mcr*-1 increased *E. coli* HS resistance to PAM and decreased inflammatory response when HT-29 cells were stimulated by LPS (Fig 7A and B, respectively). Finally, *E. coli* HS were able to better adhere to HT-29 cells when they harbor *mcr-1* gene (Fig. 7C). Altogether, our results suggest that *mcr*-1-induced modifications are not strain dependent.

### Modifications induced by mcr-1 are dependent on Mcr-1 enzymatic activity

**Fig. 8:**
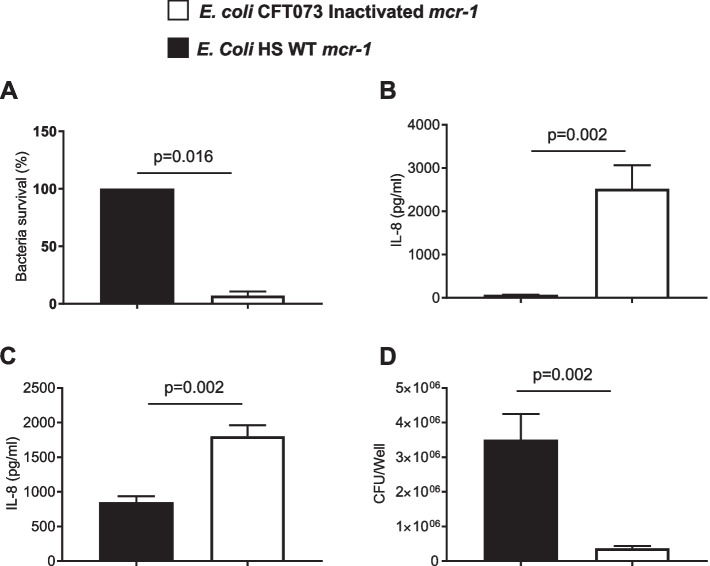
A functional Mcr-1 is required to induce (i) LL-37 resistance, (ii) modulation of pro-inflammatory properties of LPS, and (iii) increase bacterial adhesion to eukaryotic cells. *E. coli* CFT073 were transformed with wild-type *mcr-1* (WT *mcr-*1) or inactivated *mcr-1* (Inactivated *mcr-1*). **A** 2.10^7^*E. coli* WT *mcr-1*^*+*^ or 2.10^7^*E. coli* Inactivated *mcr-1*^*-*^ were incubated for 2 h with 0.2 μg of LL-37. The percentage of surviving bacteria was reported. Data are the means ± SEMs of four replicates and are representative of six independent experiments. **B**, **D** HT-29 cells were infected at an MOI of 100 for 3 h with *E. coli* WT *mcr-1*^*+*^ or with *E. coli* Inactivated *mcr-1*^*-*^. **C** HT-29 cells were stimulated for 3 h with LPS (10 μg/ml) purified from *E. coli* WT *mcr-1*^*+*^ or *E. coli* Inactivated *mcr-1*^*-*^. **B**, **C** The amounts of secreted IL-8 in the cell culture supernatant were quantified by ELISA. Data are the means ± SEMs of six replicates and are representative of two independent experiments. **D** Adherent bacteria were counted, and the data are expressed as the number of colony-forming units (CFUs)/well. Data are the means ± SEMs of six replicates and are representative of two independent experiments

Mcr-1 possesses a phosphoethanolamine transferase activity which is responsible for resistance to colistin [6]. To correlate our observations with the enzymatic activity of Mcr-1, we mutated its catalytic site. As expected, the MIC to colistin was 0.5 μg/mL for *E. coli* CFT 073 after transformation with mutated *mcr-1* (data not shown) compared to MIC = 4 μg/mL for *E. coli* CFT073 transformed with wildtype *mcr-1*, thus demonstrating that the Mcr-1 catalytic site was ineffective. Interestingly, the expression of this non-functional Mcr-1 protein did not increase bacterial resistance to PAM (Fig. 8A). Furthermore, non-functional Mcr-1 significantly increased the inflammatory response of HT-29 cells in response to bacterial infection or LPS stimulation (Fig. 8B) compared to *E. coli* CFT073 expressing the wildtype Mcr-1. Finally, bacterial adhesion to HT-29 was significantly reduced when *E. coli* CFT073 expressed the mutated Mcr-1 (Fig. 8C). Altogether, the results show that the investigated *mcr-1* bioactivities are dependent on the MCR-1 enzymatic site.

### Modifications induced by mcr-1 are a common trait shared by the mcr family

The *mcr* family is rapidly growing and to date, 10 major lineages have been identified in Proteobacteria to date, such as *E. coli*, and encode proteins, which share 30–83% amino acid identity with *mcr*-1; a phophoethanolamine transferase domain, and the lipid A modification induced by Mcr-1 is seen across the different enzymes [38]. Each *mcr* gene is mostly located on conjugative plasmids widely distributed within Enterobacterale (e.g., *E. coli* and *K. pneumoniae*) and they have also been reported within other Gram-negative bacteria, such as *Pseudomonas aeruginosa* and *Acinetobacter spp* [38]. We therefore investigated whether our observations were specific to *mcr-1* or were a common trait shared by the *mcr* family. For this purpose, *E. coli* CFT073 were transformed with a *mcr-3*-, *mcr-4*-, *mcr-5*-, or *mcr-10*-encoding plasmid and their abilities to survive the most powerful antimicrobial peptide (LL-37) and to adhere to IECs were investigated. As depicted in supplementary Figs. 11 and 12, all the *mcr* genes tested increased both bacterial survival to LL37 and bacterial adhesion to IECs compared to *E. coli* CFT073 transformed with an empty vector. Furthermore, LPS extracted from *E. coli* CFT073 transformed with a *mcr-3*-, *mcr-4*-, *mcr-5*-, or *mcr-10*-encoding plasmid were less inflammatory than LPS extracted from *E. coli* CFT073 transformed with an empty plasmid (Supplementary Figure 13).

Overall, these results suggest that the *mcr* family increased bacterial survival upon challenge from antimicrobial peptides, increased bacterial adhesion, and decreased the pro-inflammatory properties of LPS.

### mcr homologs are widespread among bacteria of the human intestinal microbiota

We assessed the prevalence of *mcr-*like genes in the human intestinal microbiota using a 9.9-million gene catalog built using metagenomic sequencing of 1267 samples [39]. Although *mcr-1* was found to be present in 27 samples of the collection, *mcr-1* homologs were not looked for at the time that the catalog was built [40]. Here, we used a 3D-based prediction method called pairwise comparative modelling (PCM) [31] to identify structural homologs of *mcr* in the 9.9-million gene catalogue. We identified 470 predictions, of which 348 were of high quality (PCM score 100% and Tm Score from Tm Align [how well the generated structure aligns with the *mcr-1* structure] ≥0.9) and were considered to be *mcr-*like genes. They shared a mean amino acidity of 24.2% with *mcr-1*. Each of the 348 *mcr-*like genes was found in at least one of the 1267 subjects, with a prevalence ranging from 0.08 to 98.7%. The mean relative abundance was 7.9 × 10^-5^ (range 5.2 × 10^-10^–1.6 × 10^-5^), with the most abundant genes being the most prevalent ones (Pearson correlation test *P* = 2.2 x 10^-16^, supplementary Fig. 14). Only 124 of the 348 (35.6%) *mcr-*like genes could be assigned to a phylum, with the Proteobacteria (*n* = 64, 51.6%) and the Bacteroidetes (*n* = 35, 28.2%) phyla being the most prevalent.

These results reveal that *mcr*-like proteins are conserved proteins of the intestinal microbial proteome and are encoded by dominant phylum, thereby suggesting that they could provide a physiological advantage in colonizing the gut and possibly a benefit for the host.

## Discussion

*E. coli* has the interesting dual characteristic of being both a widespread gut commensal bacterium of vertebrates and a versatile pathogen [41–43]. Consequently, its behavior fluctuates between commensalism and pathogenicity. While pathogenicity factors have been extensively investigated, few studies have focused on the determinants that enhance *E. coli* commensalism. Our results revealed a new facet of gut colonization by *E. coli* in which the *mcr*-1 gene enhances gut encroachment and persistence and affects several facets of *E. coli* interactions with the gut environment.

We found that *mcr*-1 and analogues enhanced *E. coli* adhesion to IECs and induced increased resistance to AMPs produced by human host cells and intestinal bacteria, which are two key functions of commensal bacteria [44–48]. *Mcr* genes did not induce resistance against nuclease AMPs, but specifically decreased the susceptibility to pore-forming AMPs targeting bacterial membrane, likewise colistin, which targets LPS, destabilizes the bacterial membrane, and consequently alters its permeability. Mcr-mediated resistance against pore-forming AMPs can therefore follow the same logic, since LPS modifications Induces by Mcr_s_ can affect the interactions of pore-forming AMPs as well as colistin with bacterial membranes.

Future therapeutic development of membrane-targeting AMPs is therefore likely to be impeded by the spread of plasmid-mediated *mcr* resistance genes. Another consequence is the probable persistence of *mcr* genes via mechanisms that are independent of therapeutic use. Accordingly, the increasing prevalence of *mcr* genes has been reported in the context of decreasing exposure to colistin [49]. If other resistance genes associated with *mcr-*1 can explain such evolution, the improvement of intestinal fitness mediated by the *mcr*-1 gene may also contribute to its spread and counterbalance the fitness cost associated with plasmid carriage. The sustained mechanism probably involves *mcr-1*-mediated LPS modifications [6, 7], which echo modifications induced by LPS phosphatases involved in the resilience of gut commensals [44].

Accordingly, we found that *mcr*-1 analogs are highly represented in the proteome of the intestinal microbiota, including highly prevalent commensal Gram-negative bacteria such as *Bacteroidetes.* Hence, *mcr-1* may promote the functional features of LPS mimicking the behaviour of LPS in commensal bacteria, and its presence in *E. coli* may be the result of convergent evolution under the selective pressure of the intestinal ecosystem.

One of the main roles of the intestinal microbiota and the immune system, which are the major actors in the gut ecosystem, is to provide resistance against the overgrowth of indigenous pathobionts and against incursion by exogenous bacteria [50–54]. We found that the presence of *mcr-1* in *E. coli* bypasses this barrier effect and promotes *E. coli* incursion without any apparent change in diversity or composition of intestinal microbiota. Thus, *mcr*-1-harboring *E. coli* can prevent immune system activation and proinflammatory responses, which result from the modification of microbiota composition [55–58].

In addition, we also found that *mcr*-1 decreased the virulence of *E. coli*, downregulated the pro-inflammatory response of IECs and macrophages to *E. coli* colonization, and thus prevented the activation of these ‘watchdogs’ of the immune system [59, 60]. This peacekeeper activity of *mcr*-1 has been observed in live bacteria, bacterial supernatants, and derivate LPS, which can diffuse from the gut lumen to the surface of IECs [36] Overall, *mcr*-1 and its analogs favor discreet incursion of *E. coli* into the intestinal ecosystem and enhance the tolerance of *E. coli* via the intestinal immune system.

*Mcr* genes have never been previously reported in intestinal-pathogenic *E. coli* (IPEC), which harbor highly specialized factors responsible for intestinal colonization, but only in commensal *E. coli* (e.g., *E. coli* phylogroups A and B1) and extra-intestinal pathogenic *E. coli* (ExPEC, phylogoup B2), which usually colonize the gut before infecting extra-intestinal sites. ExPEC, unlike IPEC, are not well defined and aggregate strains harboring heterogenous contents of pathogenicity factors contributing to gut colonization and virulence [61]. There is probably a gradient of virulence between commensal *E. coli* strains solely responsible for opportunistic extra-intestinal infections in immune-deficient hosts and highly virulent strains responsible for extra-intestinal infections in immune-competent and immune-deficient hosts. In this context, Mcr-1 may favor intestinal colonization by ExPEC and commensal strains, offset fitness cost of plasmids but probably affect their virulence and their ability to cause extra-intestinal infections and damage.

## Conclusions

The *mcr-1* gene, and probably its analogs, favors gut colonization in the absence of antibiotics. In addition to conferring resistance to colistin, they provide peacekeeper and resilience activities observed in prominent gut commensals, thereby suggesting that they enhance *E. coli* lifestyle toward intestinal commensalism. The explosive emergence of *mcr-1* is probably the consequence of colistin overuse [7]. Moreover, the presence and persistence of this gene in *E. coli,* even in the absence of antibiotics, could also be the coincidental by-product of selective pressure exerted by the intestinal ecosystem. This new paradigm in which antibiotic resistance enhances intestinal colonization in the absence of antibiotics is an additional concern in the fight against antibiotic resistance and the sustainability of colistin and related antibiotics as a last-resort treatment in infections caused by Gram-negative bacteria.

## Supplementary Information

**Additional file 1: Table S1.** Mouse microbiota strains used in this study. **Supplementary Figure 1.** Presence of *mcr-1* does not impact bacterial growth. 10^3^ bacteria were incubated in 10 mL of (A) LB, (B) DMEM, or (C) RPMI and grown at 37°C. After dilutions, bacteria were counted, and data expressed as the total number of colony forming units (CFUs)/10 mL. Data are the means ± SEMs of three replicates and are representative of three independent experiments. **Supplementary Figure 2.** Without kanamycin pressure, the loss of pBK-CMV is similar between *E. coli* CFT073 transformed with the empty plasmid or the plasmid encoding *mcr-1* . *E. coli *CFT073 transformed with the empty plasmid or the plasmid encoding *mcr-1* were cultured for 10 days without antibiotic and bacterial population containing or not the plasmid was determined. Data are the means ± SEMs of three replicates and are representative of three independent experiments. Black line represents 25% of bacterial population harbouring the pBK-CMV plasmid. **Supplementary Figure 3.** Absolute abundances of bacterial strains in the oligo-mouse-microbiota 12 (OMM^12^) model in response to gut colonization by *E. coli mcr-1*^+^ (black bar) or by *E. coli* *mcr-1*^-^ (white bar). Gnotobiotic OMM^12^ mice were orally infected with *E. coli mcr-1*^+^ or *E. coli mcr-1*^-^, and faecal samples were collected at day 10 post-infection. The absolute abundance of each bacterial strain was determined by a strain-specific qPCR assay and is plotted as 16S rRNA gene copy numbers per ng of faecal genomic DNA. Data are represented as means ± SEMs. I46, *Clostridium innocuum*; I48, *Bacteroides caecimuris*; I49, *Lactobacillus reuteri*; KB1, *Enterococcus faecalis*; YL27, *Muribaculum intestinale*; YL31, *Flavonifractor plautii*; YL32, *Clostridium clostridioforme*; YL44, *Akkermansia muciniphila*; YL45, *Turicimonas muris*, and YL58, *Blautia coccoides*. **Supplementary Figure 4.** Caecal concentration of KC, IL-1β and IL-6 (A to C) and faecal lipocalin-2 concentration (D) measured in the oligo-mouse-microbiota 12 (OMM^12^) model in response to gut colonization by *E. coli mcr-1*^+^ (black bar) or by *E. coli mcr-1*^*-*^ (white bar). Gnotobiotic OMM^12^ mice were orally infected by *E. coli mcr-1*^+^ or by *E. coli mcr-1*^-^, and cytokines as well as lipocalin-2 (Lp-2) production were assessed at day 10 post-infection by ELISA. Data are represented as the means ± SEMs. **Supplementary Figure 5.** *mcr-1* promotes colonization of the mouse intestinal tract in a competition experiment. Mice were challenged by oral gavage with *E. coli mcr-1*^+^ and with *E. coli mcr-1*^-^. The numbers of *E. coli mcr-1*^*+*^ or *E. coli mcr-1*^*-*^ were determined from the faeces at 41 day 9 post-infection. Data are represented as means ± SEMs. **Supplemental Figure 6.** *mcr-1* preserves microbiota richness. Mice were challenged by oral gavage with *E. coli mcr-1*^+^ or with *E. coli mcr-1*^-^. The composition of the intestinal microbiota was assessed from faecal 16S rRNA gene sequencing before *E. coli* inoculation and at three days post-inoculation. The differences in alpha diversity were assessed by Shannon (A), InvSimpson (B), evenness (C), and richness (D) indices. The Kruskal-Wallis test was used to estimate significant differences among groups, and pairwise comparisons were performed using the Wilcoxon test. In addition, the p-values were adjusted for multiple group comparisons by the FDR procedure. **Supplementary Figure 7.** Taxa exhibiting significant differences in abundance in the intestinal microbiota. Taxa are reported in a 16S rRNA gene-based neighbour-joining tree (A) and the differences in their abundance are shown as log2 fold changes (B). Significant differences in abundance were assessed by the Deseq2 approach. **Supplementary Figure 8.** mcr-1 reduces inflammation in mice. Mice were challenged by oral gavage with *E. coli mcr-1*^+^ (black bar) or with *E. coli mcr-1*^-^ (white bar). Pro-inflammatory cytokine levels in the colon (A) and in the ileum (B) were measured by ELISA. Data are represented as the means ± SEMs. **Supplementary Figure 9.** Absence of Camp did not impact bacterial gut colonization. Camp deficient mice were challenged by oral gavage with *E. coli mcr-1*^+^ or with *E. coli mcr-1*^-^. The numbers of *E. coli mcr-1*^+^ or *E. coli mcr-1*^-^ were determined in faeces collected at days 1 and 3 post-infection. Data are means ± SEMs. **Supplementary Figure 10.** mcr-1 decreases bacterial and LPS virulence. Mice were inoculated by intraperitoneal injection with *E. coli mcr-1*^+^, *E. coli mcr-1*^-^ (A), or LPS purified from those bacteria (B), and the survival rate was monitored. The Mantel-Cox test and the Gehan Breslow-Wilcoxon test were used to compare the resulting survival curves. **Supplemental Figure 11.** MCRs increase bacterial resistance to LL-37. 2.10^7^ *E. coli mcr-3, -4, -5,* and *-10* or 2.10^7^ isogenic *E. coli *devoid of the gene *mcr* were incubated for 2 h with LL-37. The percentage of surviving bacteria was reported. Data are the means ± SEMs of four replicates and are representative of three independent experiments. **Supplementary Figure 12.** MCRs increase bacterial adherence to eukaryotic cells. HT-29 cells were infected for 3 h with *E. coli mcr-3, -4,* *-5*, and *-10* or with an isogenic *E. coli* devoid of the gene *mcr*. Adherent bacteria were counted, and the data are expressed as the number of colony forming units (CFUs)/well. Data are the means ± SEMs of six replicates and are representative of three independent experiments. **Supplementary Figure 13.** MCRs decrease the pro-inflammatory response induced by the LPS. HT-29 cells were stimulated with 0.01 μg/ml of LPS purified from *E. coli mcr-3, -4, -5*, and *-10* or an isogenic *E. coli* devoid of gene mcr for 3 h. The amount of secreted IL-8 in the cell culture supernatant was quantified by ELISA. Data are the means ± SEMs of six replicates and are representative of three independent experiments. **Supplementary Figure 14.** The mean relative abundances of *mcr-1*-like genes in the bacterial intestinal metagenome is positively correlated with their prevalence. **Supplementary Figure 15.** The mean relative abundances of the *mcr-1*-like genes was significantly lower in the IBD group than in the control group (Wilcoxon unpaired test P = 8.2×10^-7^). The distribution of the *mcr-1*-like genes was assessed in subjects with (*n* = 148) or without (*n* = 745) inflammatory bowel disease (IBD) using previously reported data.

## Data Availability

The reads of the bacterial 16S rRNA genes were deposited in the European Nucleotide Archive (project ID: PRJEB33293).

## Abbreviations

CFU: Colony-forming units
DMEM: Dulbecco’s modified Eagle medium
LPS: Lipopolysaccharides
MIC: Minimal inhibitory concentration
MH: Mueller-Hinton medium
MOI: Multiplicity of infection
NCBI: National Centre for Biotechnology Information
OMM^12^: Oligo-mouse-microbiota 12
PBS: Phosphate-buffered saline
PMA: Phorbol myristate acetate
sDMDMm2: Stable defined moderately diverse microbiota mice
NMDS: Non-metric multidimensional scaling
