# Correction: Genes *mcr* improve the intestinal fitness of pathogenic *E. coli* and balance their lifestyle to commensalism

**DOI:** 10.1186/s40168-023-01489-y

**Published:** 2023-02-08

**Authors:** Guillaume Dalmasso, Racha Beyrouthy, Sandrine Brugiroux, Etienne Ruppé, Laurent Guillouard, Virginie Bonnin, Pierre Saint-Sardos, Amine Ghozlane, Vincent Gaumet, Nicolas Barnich, Julien Delmas, Richard Bonnet

**Affiliations:** 1 Université Clermont Auvergne, Inserm U1071, USC-INRAe 2018, Microbes, Intestin, Inflammation et Susceptibilité de l’Hôte (M2iSH), Centre de Recherche en Nutrition Humaine Auvergne, 28 place Henri Dunant, 63001 Clermont-Ferrand, France; 2 Centre de référence de la résistance aux antibiotiques, Centre Hospitalier Universitaire, 58 place Montalembert, 63000 Clermont-Ferrand, France; 3 Université de Paris, IAME, INSERM, F-75018 Paris, France; 4 AP-HP, Hôpital Bichat, DEBRC, F-75018 Paris, France; 5 Hub de Bioinformatique et Biostatistique—Département Biologie Computationnelle, Institut Pasteur, USR 3756 CNRS, Paris, France; 6 IMOST, UMR 1240 Inserm, Université Clermont Auvergne, 58 Rue Montalembert, 63005 Clermont-Ferrand, France

**Correction: Microbiome 11, 12 (2023)**

**https://doi.org/10.1186/s40168-022-01457-y**

Following publication of the original article [1], the author reported that the given names and family names of all authors were incorrectly transposed. This has been corrected in this correction article and the original article has been updated.
