# Supplementary material for: Genes mcr improve the intestinal fitness of pathogenic E. coli and balance their lifestyle to commensalism
Source: Microbiome. 2023 Jan 20;11:12. doi: 10.1186/s40168-022-01457-y (PMC9863213; doi:10.1186/s40168-022-01457-y)

Supplementary information

Table S1: Mouse microbiota strains used in this study.

Name genus/species	Strain designation	DSM number	Phylum	Family	Reference
Bifidobacterium animalis subsp. animalis	**YL2**	26074	Actinobacteria	Bifidobacteriaceae	(Brugiroux et al., 2016; Li et al., 2015)
Muribaculum intestinale	**YL27**	28989	Bacteroidetes	Muribaculaceae	(Brugiroux et al., 2016; Li et al., 2015)
Bacteroides caecimuris	**I48**	26085	Bacteroidetes	Bacteroidaceae	(Brugiroux et al., 2016; Li et al., 2015)
Turicimonas muris	**YL45**	26109	Proteobacteri a	Sutterellaceae	(Brugiroux et al., 2016; Li et al., 2015)
Akkermansia muciniphila	**YL44**	26127	Verrucomicro bia	Akkermansiaceae	(Brugiroux et al., 2016; Li et al., 2015)
Enterococcus faecalis	**KB1**	32036	Firmicutes	Enterococcaceae	(Brugiroux et al., 2016; Li et al., 2015)
Lactobacillus reuteri	**I49**	32035	Firmicutes	Lactobacillaceae	(Brugiroux et al., 2016; Li et al., 2015)
Clostridium clostridioforme	**YL32**	26114	Firmicutes	Lachnospiraceae	(Brugiroux et al., 2016; Li et al., 2015)
Blautia coccoides	**YL58**	26115	Firmicutes	Lachnospiraceae	(Brugiroux et al., 2016; Li et al., 2015)
Flavonifractor plautii	**YL31**	26117	Firmicutes	Lachnospiraceae	(Brugiroux et al., 2016; Li et al., 2015)
Acutalibacter muris	**KB18**	26090	Firmicutes	Ruminococcaceae	(Brugiroux et al., 2016; Li et al., 2015)
Clostridium innocuum	**I46**	26113	Firmicutes	Allobaculum_f	(Brugiroux et al., 2016; Li et al., 2015)

Supplementary Figure 1: Presence of *mcr-1* does not impact bacterial growth. 10^3 bacteria were incubated in 10 mL of (**A**) LB, (**B**) DMEM, or (**C**) RPMI and grown at 37°C. After dilutions, bacteria were counted, and data expressed as the total number of colony forming units (CFUs)/10 mL. Data are the means ± SEMs of three replicates and are representative of three independent experiments.

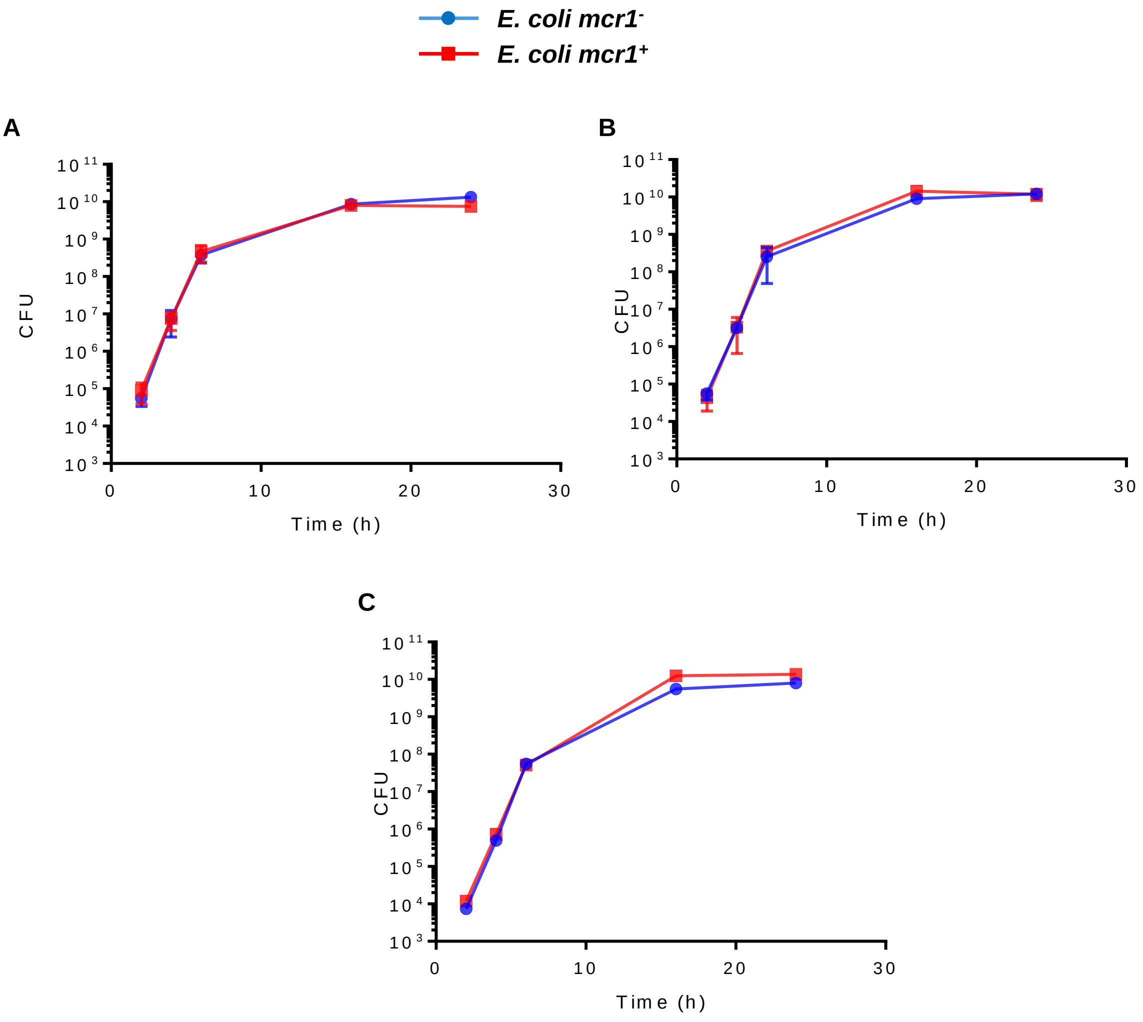

Supplementary Figure 2: Without kanamycin pressure, the loss of pBK-CMV is similar between *E. coli* CFT073 transformed with the empty plasmid or the plasmid encoding *mcr-1*. *E. coli* CFT073 transformed with the empty plasmid or the plasmid encoding *mcr-1* were cultured for 10 days without antibiotic and bacterial population containing or not the plasmid was determined. Data are the means ± SEMs of three replicates and are representative of three independent experiments. Black line represents 25% of bacterial population harbouring the pBK-CMV plasmid.

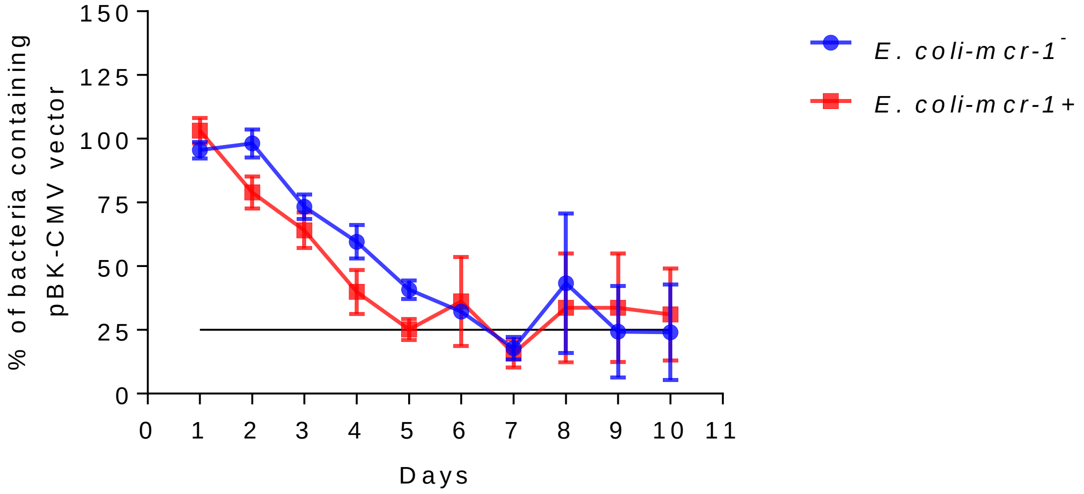

Supplementary Figure 3: Absolute abundances of bacterial strains in the oligo-mouse-microbiota 12 (OMM^{12}) model in response to gut colonization by *E. coli mcr-1^{+}* (black bar) or by *E. coli mcr-1^{-}* (white bar). Gnotobiotic OMM^{12} mice were orally infected with *E. coli mcr-1^{+}* or *E. coli mcr-1^{-},* and faecal samples were collected at day 10 post-infection. The absolute abundance of each bacterial strain was determined by a strain-specific qPCR assay and is plotted as 16S rRNA gene copy numbers per ng of faecal genomic DNA. Data are represented as means ± SEMs. I46, *Clostridium innocuum*; I48, *Bacteroides caecimuris*; I49, *Lactobacillus reuteri*; KB1, *Enterococcus faecalis*; YL27, *Muribaculum intestinale*; YL31, *Flavonifractor plautii*; YL32, *Clostridium clostridioforme*; YL44, *Akkermansia muciniphila*; YL45, *Turicimonas muris*, and YL58, *Blautia coccoides*.

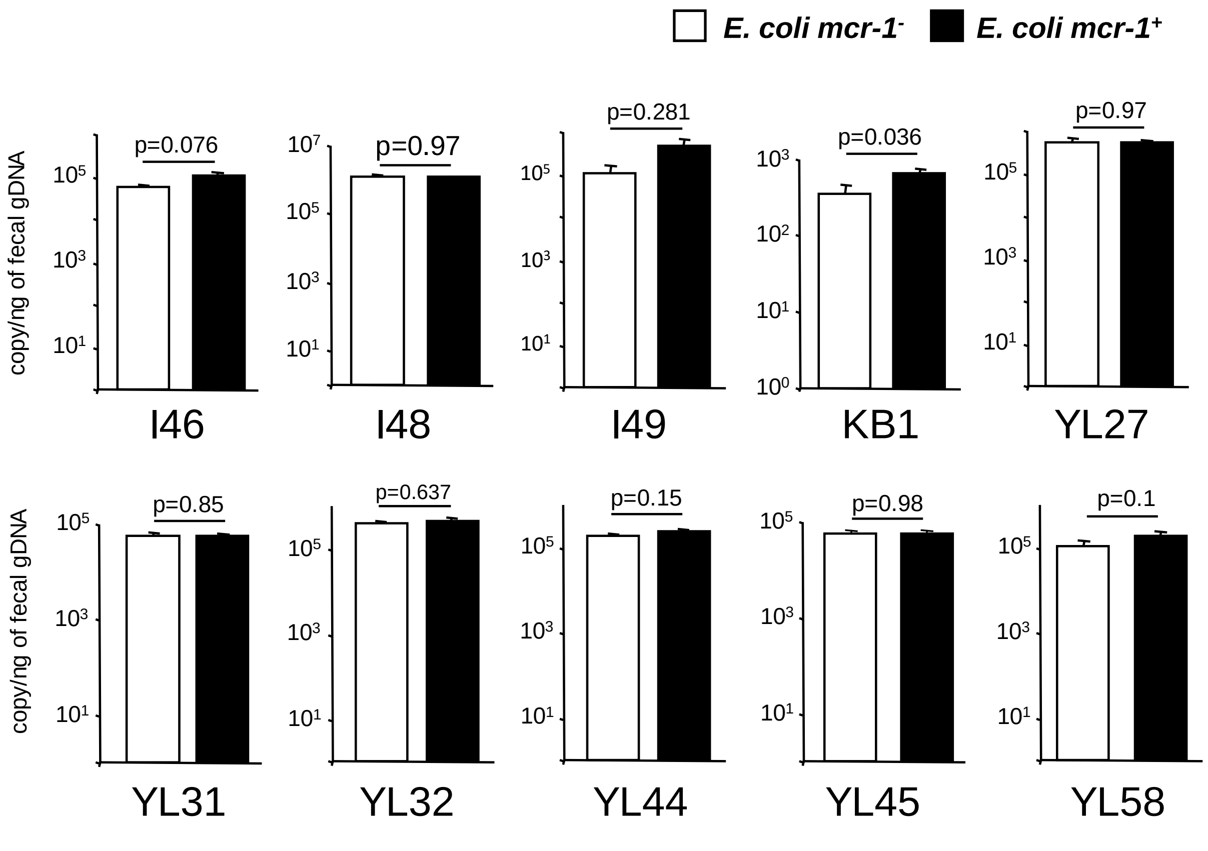

Supplementary Figure 4: Caecal concentration of KC, IL-1β and IL-6 (A to C) and faecal lipocalin-2 concentration (D) measured in the oligo-mouse-microbiota 12 (OMM^{12}) model in response to gut colonization by *E. coli mcr-1⁺* (black bar) or by *E. coli mcr-1⁻* (white bar). Gnotobiotic OMM^{12} mice were orally infected by *E. coli* $mcr\text{-}1^{+}$ or by *E. coli* $mcr\text{-}1^{-}$, and cytokines as well as lipocalin-2 (Lp-2) production were assessed at day 10 post-infection by ELISA. Data are represented as the means ± SEMs.

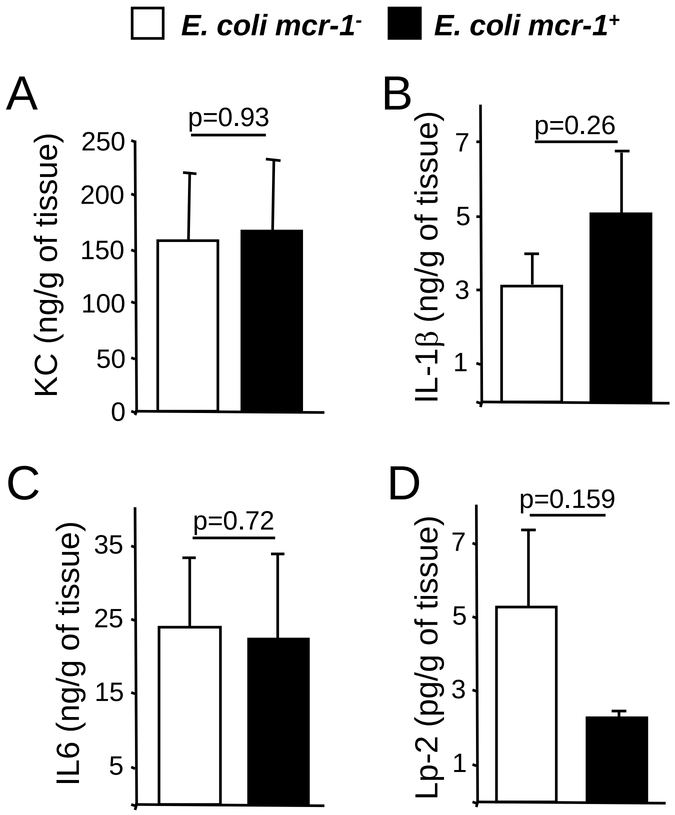

Supplementary Figure 5: *mcr-1* promotes colonization of the mouse intestinal tract in a competition experiment. Mice were challenged by oral gavage with *E. coli mcr-1⁺* and with *E. coli mcr-1⁻*. The numbers of *E. coli mcr-1⁺* or *E. coli mcr-1⁻* were determined from the faeces at day 9 post-infection. Data are represented as means ± SEMs.

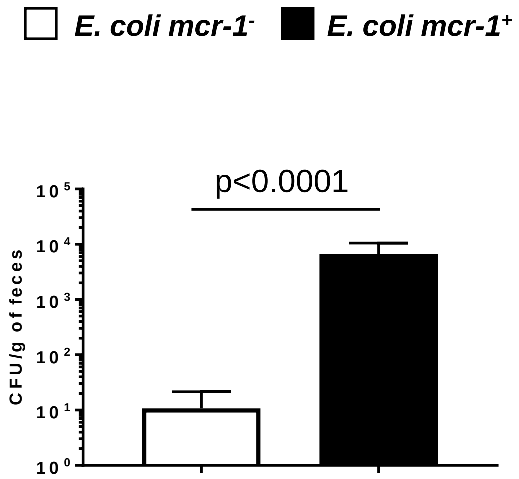

Supplemental Figure 6: *mcr-1* preserves microbiota richness. Mice were challenged by oral gavage with *E. coli mcr-1*$^{+}$ or with *E. coli mcr-1*$^{-}$. The composition of the intestinal microbiota was assessed from faecal 16S rRNA gene sequencing before *E. coli* inoculation and at three days post-inoculation. The differences in alpha diversity were assessed by Shannon (**A**), InvSimpson (**B**), evenness (**C**), and richness (**D**) indices. The Kruskal-Wallis test was used to estimate significant differences among groups, and pairwise comparisons were performed using the Wilcoxon test. In addition, the *p*-values were adjusted for multiple group comparisons by the FDR procedure.

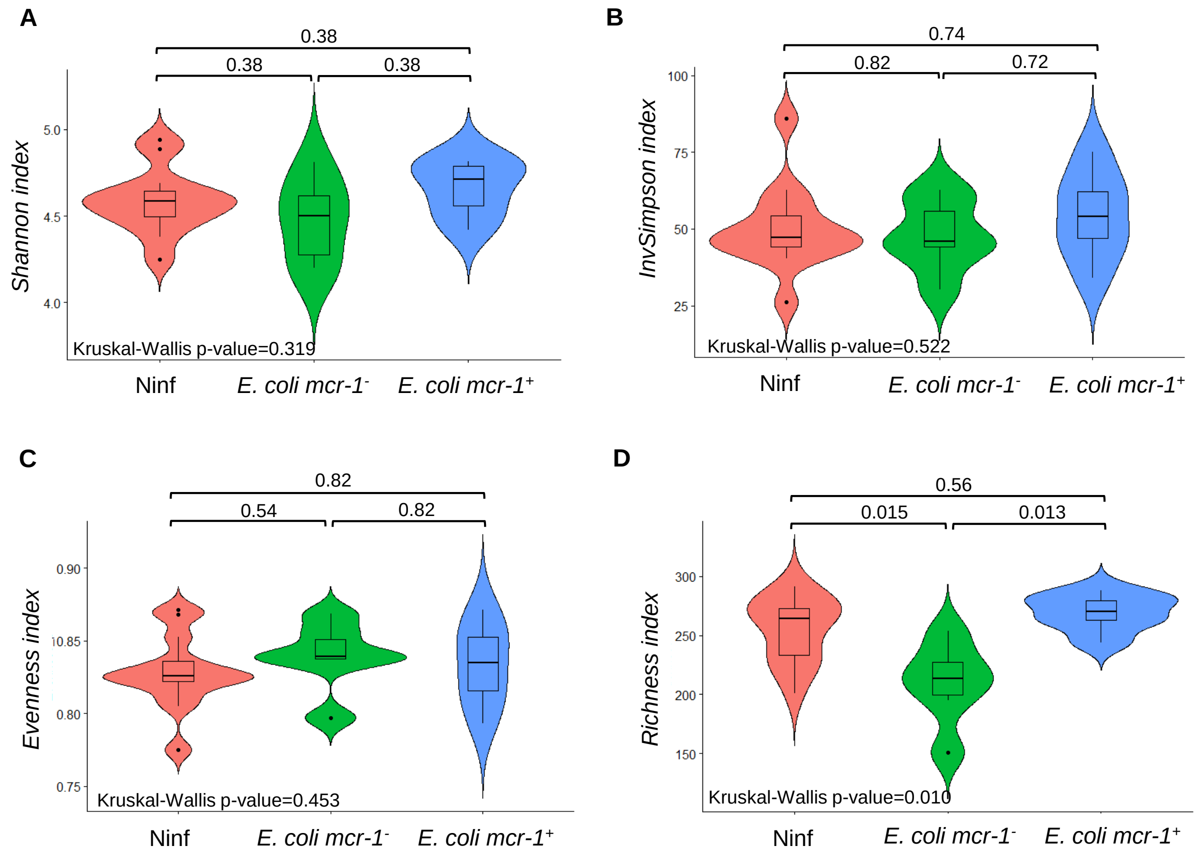

Supplementary Figure 7: Taxa exhibiting significant differences in abundance in the intestinal microbiota. Taxa are reported in a 16S rRNA gene-based neighbour-joining tree (**A**) and the differences in their abundance are shown as $\log_2$ fold changes (**B**). Significant differences in abundance were assessed by the Deseq2 approach.

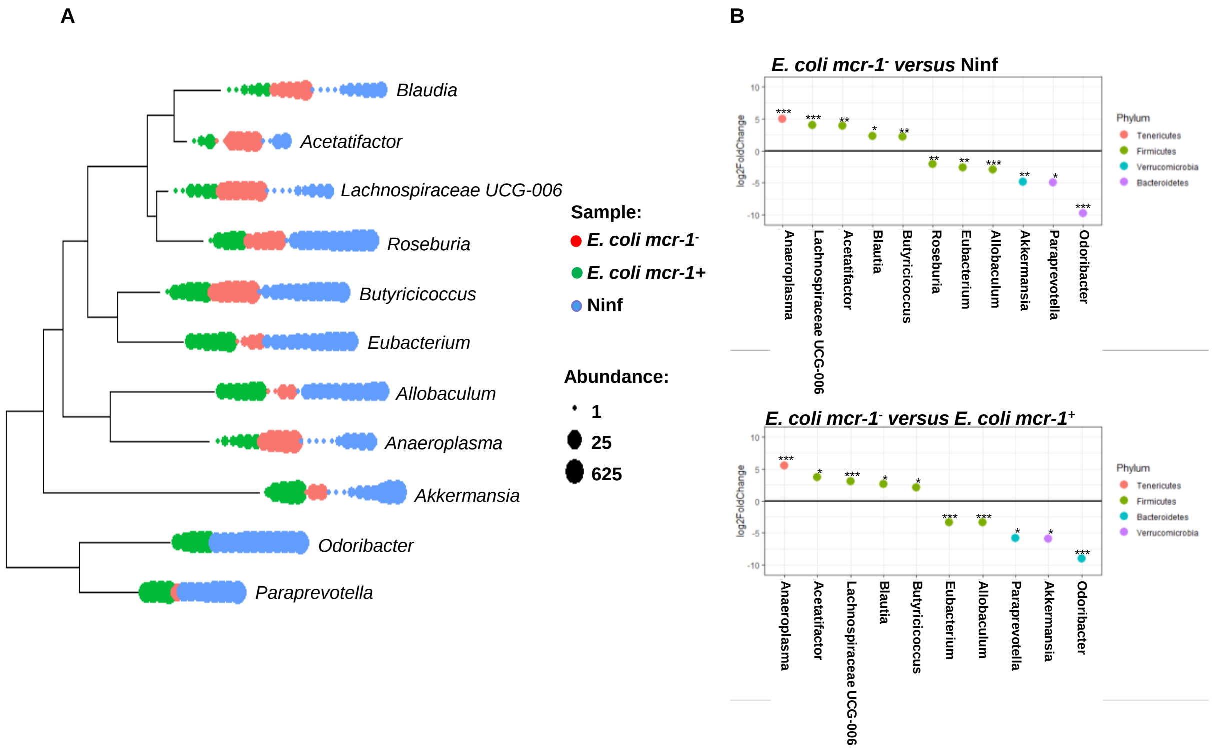

Supplementary Figure 8: *mcr-1* reduces inflammation in mice. Mice were challenged by oral gavage with *E. coli mcr-1*$^{+}$ (black bar) or with *E. coli mcr-1*$^{-}$ (white bar). Pro-inflammatory cytokine levels in the colon (**A**) and in the ileum (**B**) were measured by ELISA. Data are represented as the means ± SEMs.

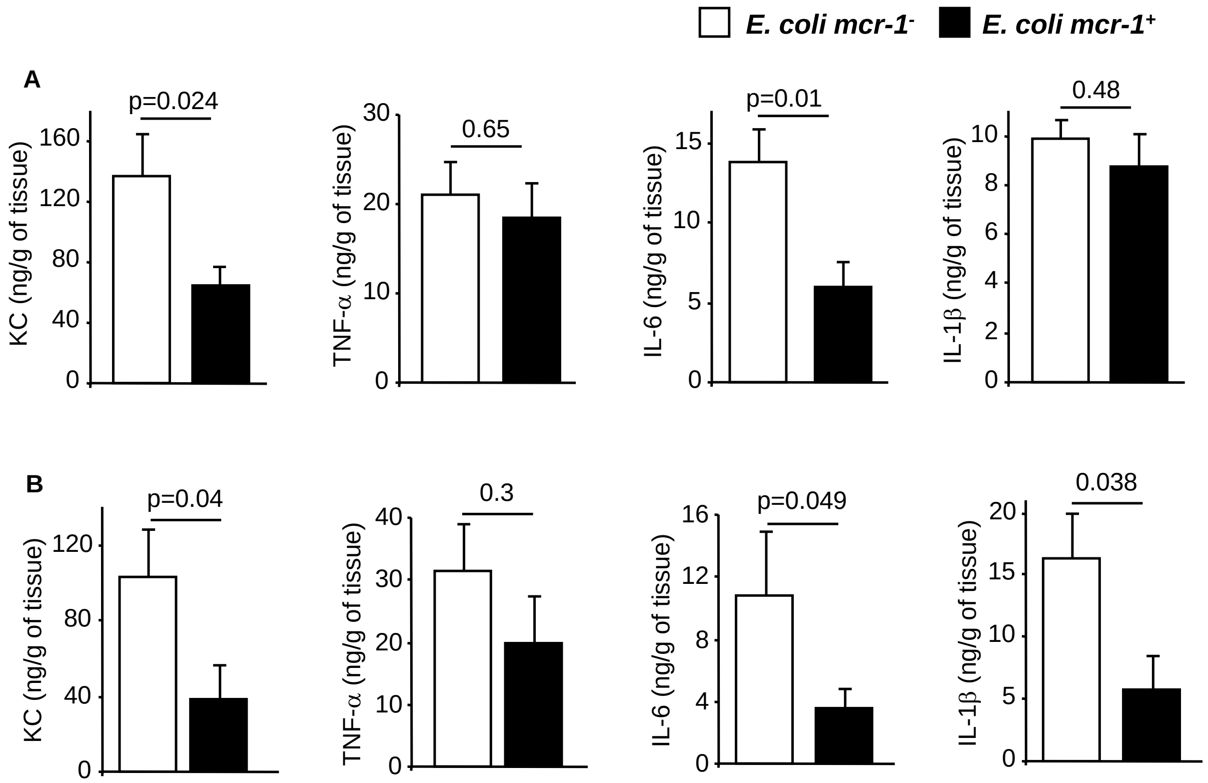

Supplementary Figure 9. Absence of *Camp* did not impact bacterial gut colonization. *Camp* deficient mice were challenged by oral gavage with *E. coli mcr-1*$^{+}$ or with *E. coli mcr-1*$^{-}$. The numbers of *E. coli mcr-1*$^{+}$ or *E. coli mcr-1*$^{-}$ were determined in faeces collected at days 1 and 3 post-infection. Data are means ± SEMs.

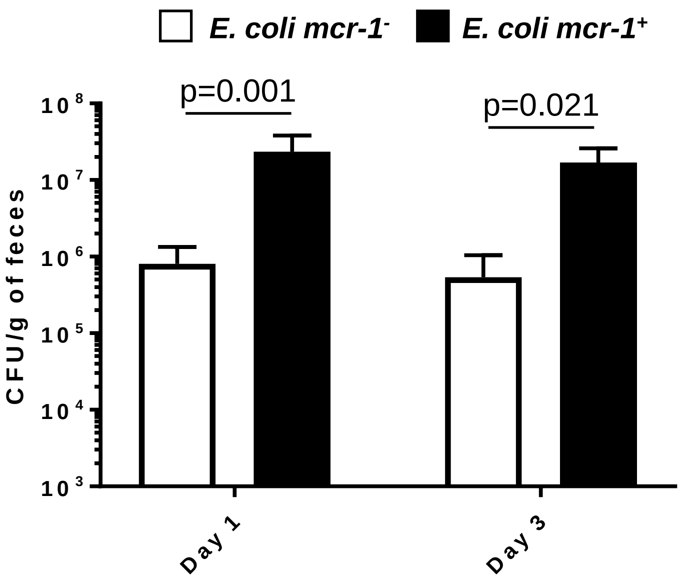

Supplementary Figure 10: *mcr-1* decreases bacterial and LPS virulence. Mice were inoculated by intraperitoneal injection with *E. coli mcr-1*$^{+}$, *E. coli mcr-1*$^{-}$ (**A**), or LPS purified from those bacteria (**B**), and the survival rate was monitored. The Mantel-Cox test and the Gehan-Breslow-Wilcoxon test were used to compare the resulting survival curves.

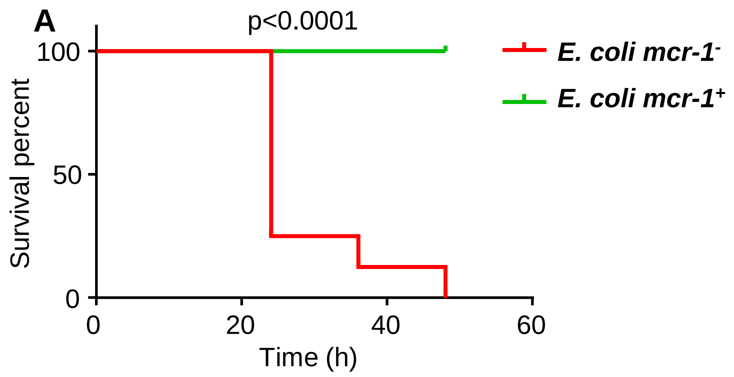

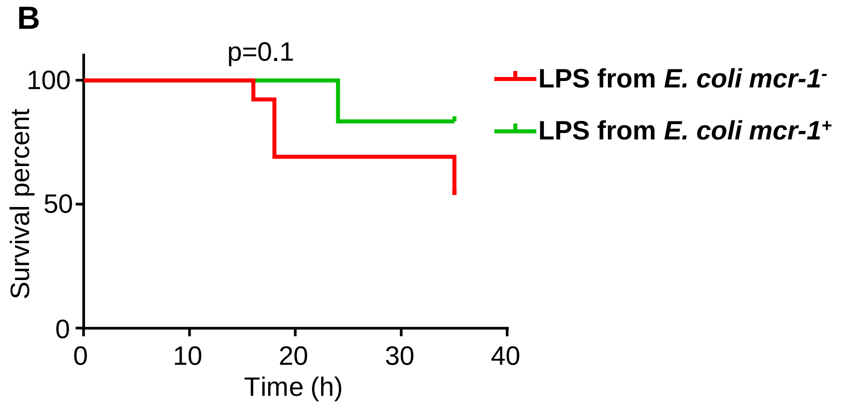

Supplemental Figure 11. MCRs increase bacterial resistance to LL-37. 2.10^7 *E. coli mcr-3*, *-4*, *-5*, and *-10* or 2.10^7 isogenic *E. coli* devoid of the gene *mcr* were incubated for 2 h with LL-37. The percentage of surviving bacteria was reported. Data are the means ± SEMs of four replicates and are representative of three independent experiments.

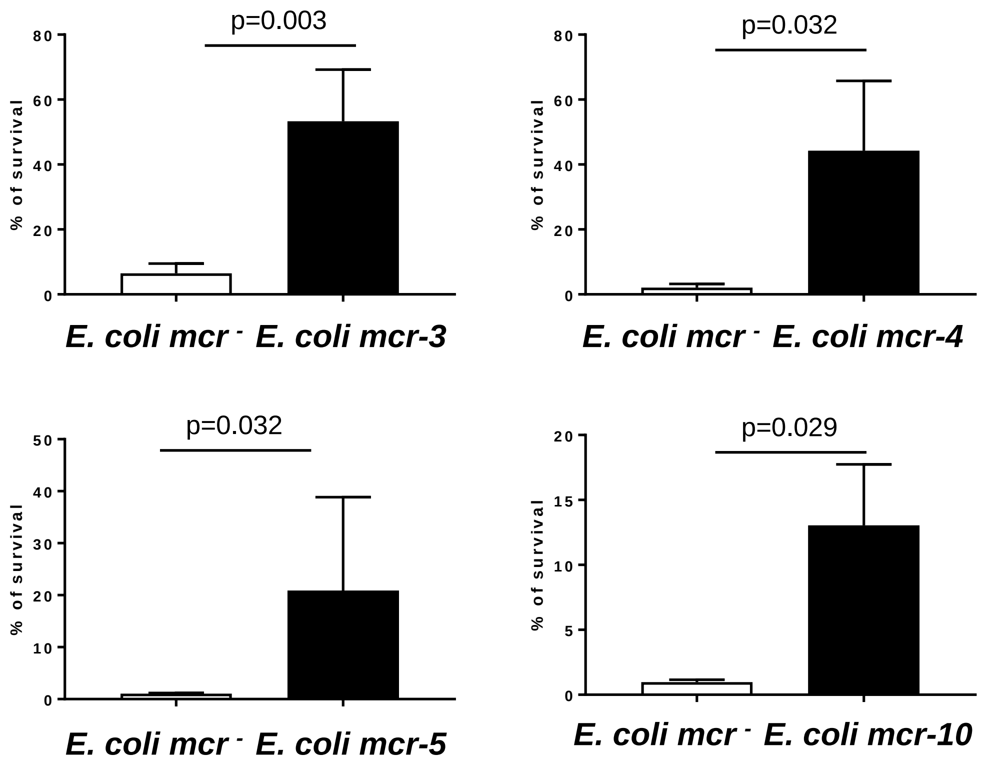

Supplementary Figure 12. MCRs increase bacterial adherence to eukaryotic cells. HT-29 cells were infected for 3 h with *E. coli mcr-3*, *-4*, *-5*, and *-10* or with an isogenic *E. coli* devoid of the gene *mcr*. Adherent bacteria were counted, and the data are expressed as the number of colony forming units (CFUs)/well. Data are the means ± SEMs of six replicates and are representative of three independent experiments.

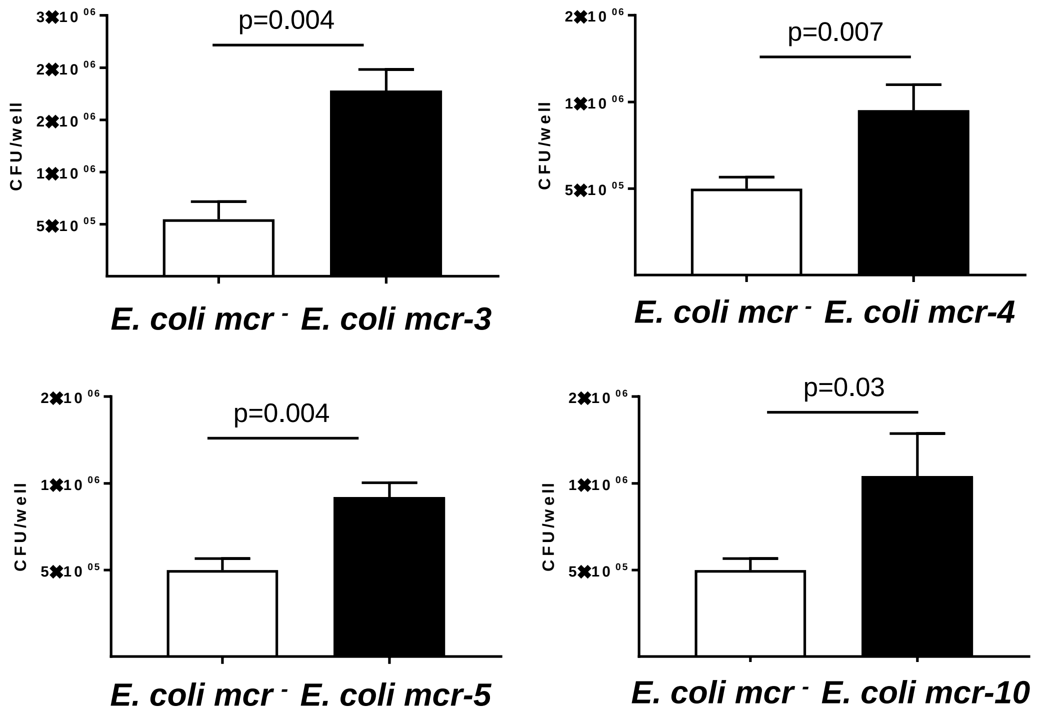

Supplementary Figure 13. MCRs decrease the pro-inflammatory response induced by the LPS. HT-29 cells were stimulated with 0.01 µg/ml of LPS purified from *E. coli mcr-3*, *-4*, *-5,* and *-10* or an isogenic *E. coli* devoid of gene *mcr* for 3 h. The amount of secreted IL-8 in the cell culture supernatant was quantified by ELISA. Data are the means ± SEMs of six replicates and are representative of three independent experiments.

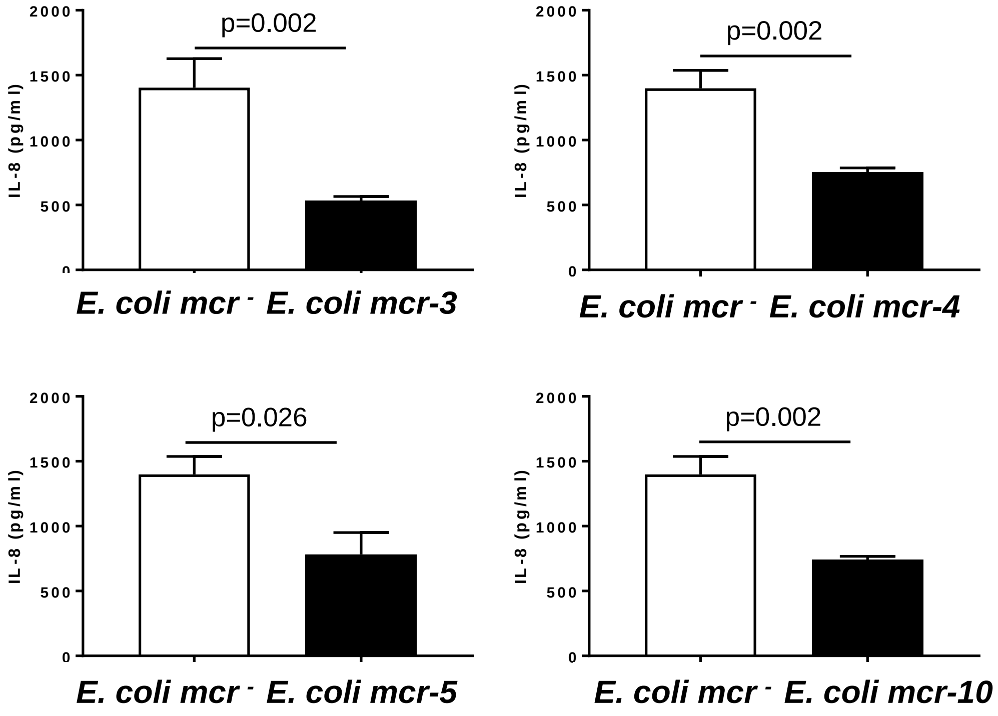

Supplementary Fig. 14: The mean relative abundances of *mcr-1*-like genes in the bacterial intestinal metagenome is positively correlated with their prevalence.

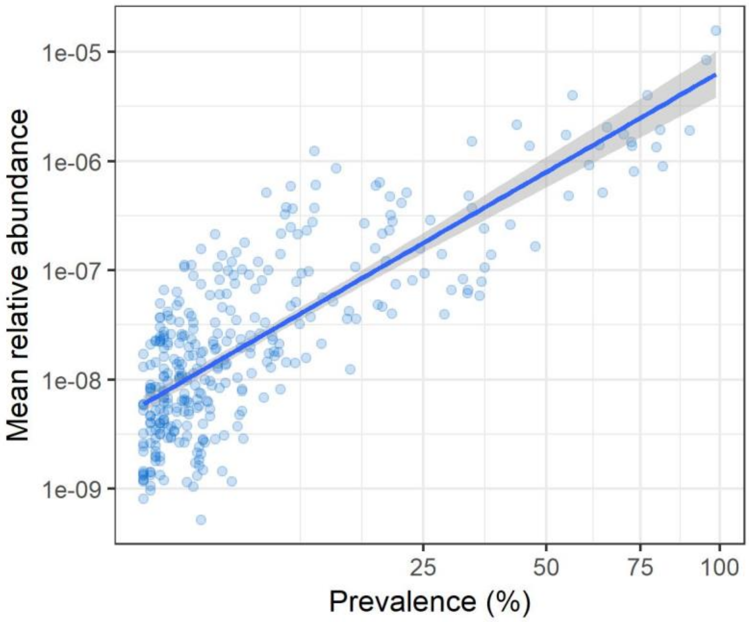

Supplementary Figure 15: The mean relative abundances of the *mcr-1*-like genes was significantly lower in the IBD group than in the control group (Wilcoxon unpaired test $P = 8.2\times10^{-7}$). The distribution of the *mcr-1*-like genes was assessed in subjects with (n = 148) or without (n = 745) inflammatory bowel disease (IBD) using previously reported data.[25]

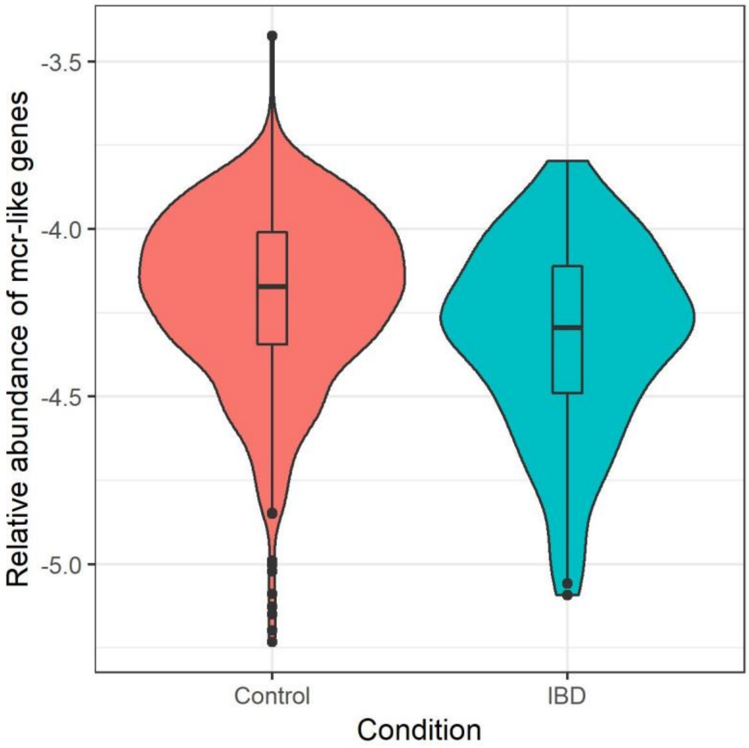

Supplement: Supplementary file 2 — Additional file 1: Table S1. Mouse microbiota strains used in this study. Supplementary Figure 1. Presence of mcr-1 does not impact bacterial growth. 103 bacteria were incubated in 10 mL of (A) LB, (B) DMEM, or (C) RPMI and grown at 37°C. After dilutions, bacteria were counted, and data expressed as the total number of colony forming units (CFUs)/10 mL. Data are the means ± SEMs of three replicates and are representative of three independent experiments. Supplementary Figure 2. Without kanamycin pressure, the loss of pBK-CMV is similar between E. coli CFT073 transformed with the empty plasmid or the plasmid encoding mcr-1 . E. coli CFT073 transformed with the empty plasmid or the plasmid encoding mcr-1 were cultured for 10 days without antibiotic and bacterial population containing or not the plasmid was determined. Data are the means ± SEMs of three replicates and are representative of three independent experiments. Black line represents 25% of bacterial population harbouring the pBK-CMV plasmid. Supplementary Figure 3. Absolute abundances of bacterial strains in the oligo-mouse-microbiota 12 (OMM12) model in response to gut colonization by E. coli mcr-1+ (black bar) or by E. coli mcr-1- (white bar). Gnotobiotic OMM12 mice were orally infected with E. coli mcr-1+ or E. coli mcr-1-, and faecal samples were collected at day 10 post-infection. The absolute abundance of each bacterial strain was determined by a strain-specific qPCR assay and is plotted as 16S rRNA gene copy numbers per ng of faecal genomic DNA. Data are represented as means ± SEMs. I46, Clostridium innocuum; I48, Bacteroides caecimuris; I49, Lactobacillus reuteri; KB1, Enterococcus faecalis; YL27, Muribaculum intestinale; YL31, Flavonifractor plautii; YL32, Clostridium clostridioforme; YL44, Akkermansia muciniphila; YL45, Turicimonas muris, and YL58, Blautia coccoides. Supplementary Figure 4. Caecal concentration of KC, IL-1β and IL-6 (A to C) and faecal lipocalin-2 concentration (D) measured in the ol [file 40168_2022_1457_MOESM1_ESM.pdf]
